# The efficacy of Ranolazine on E1784K is altered by temperature and calcium

**DOI:** 10.1038/s41598-018-22033-1

**Published:** 2018-02-26

**Authors:** Mena Abdelsayed, Manpreet Ruprai, Peter C. Ruben

**Affiliations:** Department of Biomedical Physiology and Kinesiology, Simon Fraser University, 8888 University Drive, Burnaby, BC V5A 1S6 Canada

## Abstract

E1784K is the most common mixed syndrome *SCN5a* mutation underpinning both Brugada syndrome type 1 (BrS1) and Long-QT syndrome type 3 (LQT3). The charge reversal mutant enhances the late sodium current (I_Na_) passed by the cardiac voltage-gated sodium channel (Na_V_1.5), delaying cardiac repolarization. Exercise-induced triggers, like elevated temperature and cytosolic calcium, exacerbate E1784K late I_Na_. In this study, we tested the effects of Ranolazine, the late I_Na_ blocker, on voltage-dependent and kinetic properties of E1784K at elevated temperature and cytosolic calcium. We used whole-cell patch clamp to measure I_Na_ from wild type and E1784K channels expressed in HEK293 cells. At elevated temperature, Ranolazine attenuated gain-of-function in E1784K by decreasing late I_Na_, hyperpolarizing steady-state fast inactivation, and increasing use-dependent inactivation. Both elevated temperature and cytosolic calcium hampered the capacity of Ranolazine to suppress E1784K late I_Na_. *In-silico* action potential (AP) simulations were done using a modified O’Hara Rudy (ORd) cardiac model. Simulations showed that Ranolazine failed to shorten AP duration, an effect augmented at febrile temperatures. The drug-channel interaction is clearly affected by external triggers, as reported previously with ischemia. Determining drug efficacy under various physiological states in *SCN5a* cohorts is crucial for accurate management of arrhythmias.

## Introduction

The alpha subunit of the cardiac voltage-gated sodium channel, Na_V_1.5, is encoded by the *SCN5a* gene. Mutations in this gene usually cause long-QT syndrome type 3 (LQT3), Brugada syndrome type 1 (BrS1), or both (mixed syndromes)^1–5^. These clinical conditions are elicited by expression of gating dysfunctions in Na_V_1.5^6–10^. Gain- and loss-of-function mutations can modify the inward sodium current (I_Na_). Gain-of-function (GoF) in Na_V_1.5 arises from loss in channel fast inactivation, thereby increasing the non-inactivating, late I_Na_, underlying LQT3^7,11–16^. Loss-of-function (LoF) mainly arises from decreased peak I_Na_ resulting in BrS1^1,6,17–20^. Interestingly, both GoF and LoF defects can occur simultaneously in a number of mutants^6,12,17,21–24^.

A guanine to an adenine substitution at position 5349 in *SCN5a* expresses the charge reversal mutant, E1784K, in the Na_V_1.5 C-terminal domain (CTD)^25,26^. E1784K is the most common mixed syndrome mutant, particularly prevalent in the Okinawa Islands in Japan, where carriers mainly express diagnostic LQT3^27^. Clinical studies reveal differential phenotypic expressivity in E1784K cohorts^12,22,28,29^.

**Figure 1:**
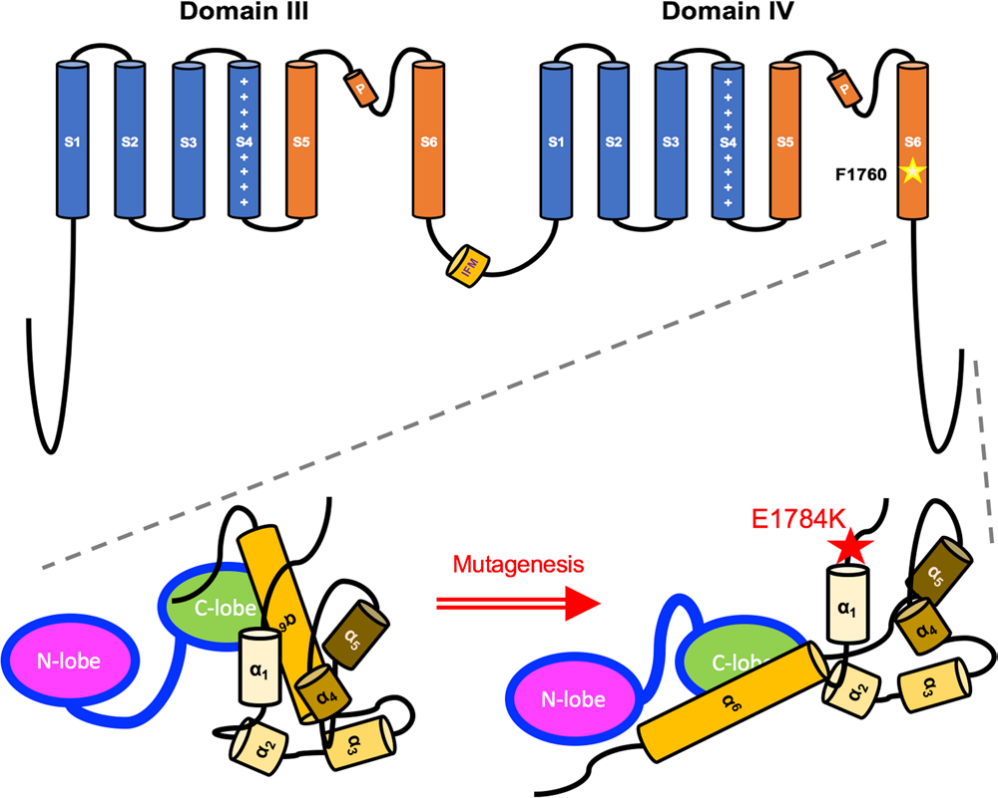
Sodium Channel Schematic Diagram. Domain III and Domain IV, along with their inter-linker and the CTD, regulate channel function and calcium sensitivity. DIII-DIV linker contains the fast inactivation “particle” (IFM motif). DIV-S6 contains the putative drug-binding residue, F1760, which is key for Ranolazine binding. The enlarged CTD contains six α-helices which aggregate to form an intact domain for calmodulin binding. Calmodulin (CaM) binds to the IQ-domain (α_6_) under low cytosolic calcium conditions (structure adapted from Chagot *et al*., 2009 and Gabelli *et al*., 2014). E1784K may rearrange CTD structure affecting Calmodulin interaction with other channel sites, such as the DIII-DIV linker.

E1784 is located directly upstream of the acidic globular EF-like hand domain (α_1_–α_4_). The residue contributes to the electrostatic interactions formed between the acidic domain and the downstream basic IQ domain (α_6_, Fig. 1 compares WT to E1784K structure)^30–34^. The proximal CTD, in which E1784K resides, has the largest effects on kinetics and steady-state inactivation^30,33,35,36^. The charge reversal mutant, E1784K, is thought to disturb the integrity of CTD, causing the α_6_ to become more mobile (Fig. 1)^37^. A disturbance to α_6_ integrity has been correlated with elevations in late I_Na_ and enhanced slow inactivation^16,35,38–40^, which are key biophysical attributes in E1784K^12,22,28^.

Recent studies characterized the effects of exercise-induced triggers on E1784K. These triggers include acidosis, elevated temperatures, and cytosolic calcium. Acidosis and elevated temperatures augment late I_Na_ and decrease peak I_Na_ in E1784K^41–43^. Use-dependence in E1784K is reduced with high stimulation frequencies at elevated temperatures^41^. Compared to other mutants, E1784K tends to hamper the native potency of cytosolic calcium to block late I_Na_ in Na_V_1.5^37,44^. Elevated cytosolic calcium augments channel availability in E1784K by depolarizing the voltage-dependence of slow inactivation^37^. Dynamic *in silico* action potential (AP) simulations in cardiac cells show E1784K-induced alternans at sinus rhythm and with tachycarida^37,41^.

We hypothesize that Ranolazine, which preferentially blocks late I_Na_, is suitable for ameliorating the thermal and calcium-induced defects in E1784K. Although prescribed as an anti-anginal drug for diastolic dysfunction treatment^45–47^, Ranolazine has anti-arrhythmic efficacy proven to be useful in treating *SCN5a* inherited conditions^48–51^. Ranolazine efficacy is enhanced with *SCN5a* mutations or channel triggers, such as acidosis, which augment late I_Na_^48,49,52–54^.We predicted that the channel mutation-trigger interaction may alter drug efficacy. Our goal is to study the effects of Ranolazine on E1784K under conditions of elevated temperature and cytosolic calcium levels.

## Results

### Ranolazine binds to NaV1.5 inner vestibule

**Figure 2:**
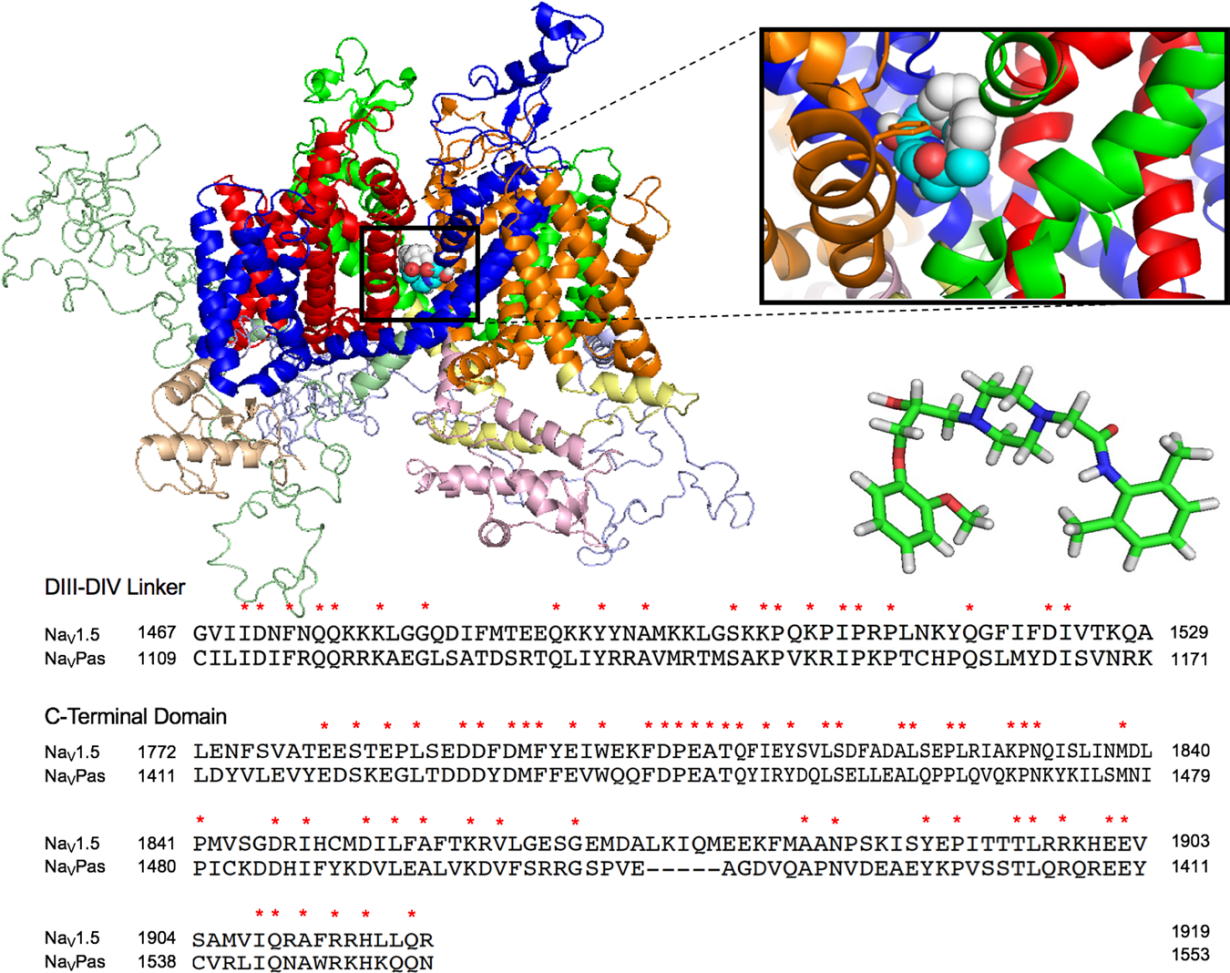
Ranolazine docked to Na_V_1.5-Na_V_Pas. The side view of Na_V_1.5-Na_V_Pas homology model is shown docked to Ranolazine. The enlarged inset shows the cartoon structure of the drug binding to the central domains of the channel. The aromatic F1760 residue is outlined. Below the inset is a 3D-structure of Ranolazine (Nitrogen is blue, Oxygen is red, Carbon is green, and Hydrogen is grey). Conserved residues in DIII-DIV linker and CTD between Na_V_1.5 and Na_V_Pas are indicated by a red asterisk.

The Na_V_1.5 homology model based on Na_V_Pas (Na_V_1.5-Na_V_Pas) is shown in Fig. 2. The side view of the channel shows the four domains and their putative voltage and pore-forming segments (including the p-helices, extracellular and intracellular linkers). Na_V_Pas shares about 32% sequence identity with Na_V_1.5. The aligned sequences for DIII-DIV linker and CTD are shown in Fig. 2. Ranolazine was auto-docked against Na_V_1.5-Na_V_Pas using AutoDock4. The highest affinity (−7.7 kcal/mol) binding mode is enlarged in Fig. 2. The compound formed polar and Van der Waals interaction with various residues located in all four domains: S401, V405, C896, N927, F1418, S1458, L1462, N1463, I1466, F1760, V1764, I1768. The aromatic residue, F1760, is outlined in Fig. 2 as it is a key putative binding site for many anti-arrhythmics, local anesthetics, and anticonvulsants^52,55^. F1760 orientation with respect to Ranolazine supports its critical role in drug binding.

### Ranolazine does not affect conductance

**Figure 3:**
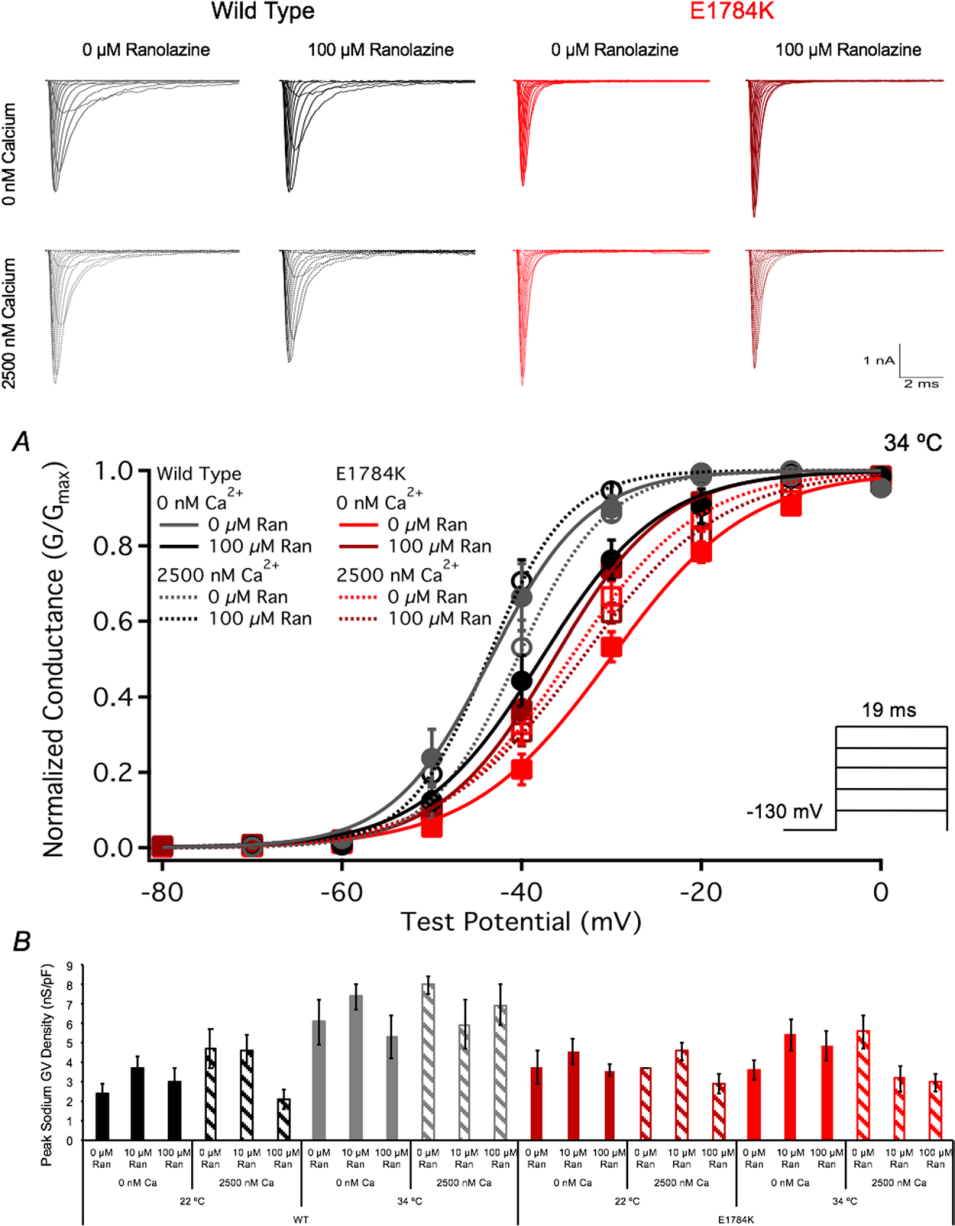
Ranolazine effects on channel conductance. Top traces show raw I_Na_ traces recorded at 34 °C. Panel A shows normalized conductance plotted against the test potential (pulse protocol shown in *inset*) at 34 °C. Panel C shows the peak conductance density bar graph versus all conditions at both 22 °C and 34 °C.

Raw current traces in Fig. 3 show the effects of 0 µM and 100 µM Ranolazine on WT and E178K at 0 nM and 2500 nM cytosolic calcium (only 34 °C shown). E1784K reduced (p < 0.0001) the peak current and conductance density compared to WT. Elevated temperature (34 °C) increased (p < 0.0001) peak current and conductance density in WT but not in E1784K. Ranolazine had no effect (p > 0.05) on peak current or conductance density (Table 1 and Fig. 3B).

**Table 1:** Peak I_Na_ Current and Conductance Density.

Condition	Peak I_Na_ Density (pA/pF)	N	Peak GV Density (nS/pF)	N
WT - 22 °C - 0 nM Ca^2+^ - 0 µM Ran	148.97 ± 35.10	7	2348.13 ± 584.87	7
WT - 22 °C - 0 nM Ca^2+^ - 10 µM Ran	241.31 ± 41.80	5	3708.29 ± 580.79	5
WT - 22 °C - 0 nM Ca^2+^ - 100 µM Ran	184.16 ± 40.16	5	3030.10 ± 640.76	5
WT - 22 °C - 2500 nM Ca^2+^ - 0 µM Ran	302.91 ± 59.07	9	4714.99 ± 971.64	9
WT - 22 °C - 2500 nM Ca^2+^ - 10 µM Ran	250.88 ± 45.34	7	4599.97 ± 788.18	7
WT - 22 °C - 2500 nM Ca^2+^ - 100 µM Ran	128.74 ± 34.69	9	2088.79 ± 517.47	9
WT - 34 °C - 0 nM Ca^2+^ - 0 µM Ran	354.74 ± 83.12	7	6067.75 ± 1197.96	6
WT - 34 °C - 0 nM Ca^2+^ - 10 µM Ran	472.24 ± 55.47	5	7370.72 ± 654.58	7
WT - 34 °C - 0 nM Ca^2+^ - 100 µM Ran	369.56 ± 81.13	8	5321.49 ± 1074.25	8
WT - 34 °C - 2500 nM Ca^2+^ - 0 µM Ran	535.63 ± 70.44	6	7979.56 ± 433.11	5
WT - 34 °C - 2500 nM Ca^2+^ - 10 µM Ran	447.46 ± 106.69	6	5948.51 ± 1253.7	6
WT - 34 °C - 2500 nM Ca^2+^ - 100 µM Ran	599.72 ± 113.81	6	6935.36 ± 1048.34	5
EK - 22 °C - 0 nM Ca^2+^ - 0 µM Ran	214.56 ± 50.18	7	3762.09 ± 835.36	7
EK - 22 °C - 0 nM Ca^2+^ - 10 µM Ran	265.65 ± 51.36	8	4534.15 ± 651.10	10
EK - 22 °C - 0 nM Ca^2+^ - 100 µM Ran	240.86 ± 24.43	7	3576.84 ± 301.37	7
EK - 22 °C - 2500 nM Ca^2+^ - 0 µM Ran	170.41 ± 31.44	5	3660.43 ± 1006.25	6
EK - 22 °C - 2500 nM Ca^2+^ - 10 µM Ran	250.36 ± 33.96	6	4562.87 ± 453.93	8
EK - 22 °C - 2500 nM Ca^2+^ - 100 µM Ran	183.02 ± 44.18	5	2886.72 ± 498.80	5
EK - 34 °C - 0 nM Ca^2+^ - 0 µM Ran	257.99 ± 34.07	8	3613.63 ± 524.71	9
EK - 34 °C - 0 nM Ca^2+^ - 10 µM Ran	322.26 ± 47.45	6	5422.63 ± 833.24	8
EK - 34 °C - 0 nM Ca^2+^ - 100 µM Ran	278.25 ± 67.75	8	4834.84 ± 766.20	7
EK - 34 °C - 2500 nM Ca^2+^ - 0 µM Ran	354.25 ± 64.03	8	5587.74 ± 831.96	7
EK - 34 °C - 2500 nM Ca^2+^ - 10 µM Ran	227.98 ± 41.82	5	3154.90 ± 685.41	5
EK - 34 °C - 2500 nM Ca^2+^ - 100 µM Ran	212.41 ± 28.73	9	2959.51 ± 471.73	9

Figure 3A shows normalized conductance plotted against the test potential at 34 °C. E1784K (p < 0.0001) and elevated temperature (p = 0.0003) depolarized the conductance midpoint (GV-V_1/2_). The conductance slope (GV-z) was reduced (p < 0.0001) in E1784K compared to WT and increased (p < 0.0001) when temperature was elevated in both channel variants. The interaction between channel variant and temperature had no effect on GV-V_1/2_ and GV-z. Normalized conductance was unchanged in all conditions with Ranolazine (Table 2).

**Table 2:** Activation and Steady-State Fast Inactivation.

Condition	GV-V_1/2_ (mV)	GV-z	N	SSFI-V_1/2_ (mV)	SSFI-z	N
WT - 22 °C - 0 nM Ca^2+^ - 0 µM Ran	−40.45 ± 1.24	3.76 ± 0.33	7	−88.62 ± 1.99	−3.93 ± 0.26	7
WT - 22 °C - 0 nM Ca^2+^ - 10 µM Ran	−45.59 ± 1.09	5.37 ± 0.29	6	−86.51 ± 1.36	−3.95 ± 0.16	7
WT - 22 °C - 0 nM Ca^2+^ - 100 µM Ran	−44.13 ± 3.07	4.84 ± 0.56	5	−88.45 ± 3.18	−2.87 ± 0.11^*2^	6
WT - 22 °C - 2500 nM Ca^2+^ - 0 µM Ran	−42.22 ± 1.16	4.08 ± 0.25	9	−91.72 ± 1.72	−3.68 ± 0.15	9
WT - 22 °C - 2500 nM Ca^2+^ - 10 µM Ran	−44.40 ± 2.22	3.87 ± 0.28	7	−91.44 ± 1.63	−3.93 ± 0.18	7
WT - 22 °C - 2500 nM Ca^2+^ - 100 µM Ran	−44.76 ± 2.09	4.38 ± 0.36	9	−90.94 ± 1.64	−2.20 ± 0.13^*2^	7
WT - 34 °C - 0 nM Ca^2+^ - 0 µM Ran	−43.44 ± 2.06	6.00 ± 0.45	6	−80.31 ± 1.17	−4.72 ± 0.18	6
WT - 34 °C - 0 nM Ca^2+^ - 10 µM Ran	−37.93 ± 1.97	4.95 ± 0.28	7	−82.51 ± 2.26	−4.00 ± 0.14	8
WT - 34 °C - 0 nM Ca^2+^ - 100 µM Ran	−37.58 ± 2.03	4.43 ± 0.33	7	−87.30 ± 2.73	−2.82 ± 0.16^*2^	7
WT - 34 °C - 2500 nM Ca^2+^ - 0 µM Ran	−40.62 ± 1.44	5.96 ± 0.35	6	−80.25 ± 2.30	−4.24 ± 0.08	7
WT - 34 °C - 2500 nM Ca^2+^ - 10 µM Ran	−38.03 ± 3.69	5.15 ± 0.40	6	−83.18 ± 3.95	−4.00 ± 0.13	6
WT - 34 °C - 2500 nM Ca^2+^ - 100 µM Ran	−43.69 ± 0.98	6.30 ± 0.48	6	−90.43 ± 2.80	−3.18 ± 0.2^*2^	6
EK - 22 °C - 0 nM Ca^2+^ - 0 µM Ran	−35.13 ± 2.70	2.99 ± 0.27	8	−100.46 ± 1.51	−2.97 ± 0.08	8
EK - 22 °C - 0 nM Ca^2+^ - 10 µM Ran	−35.83 ± 1.65	3.14 ± 0.27	10	−99.37 ± 1.61	−2.88 ± 0.08	10
EK - 22 °C - 0 nM Ca^2+^ - 100 µM Ran	−36.16 ± 1.87	3.95 ± 0.17	7	−103.83 ± 2.42	−1.77 ± 0.06^*2^	8
EK - 22 °C - 2500 nM Ca^2+^ - 0 µM Ran	−39.33 ± 2.02	3.09 ± 0.16	5	−101.30 ± 3.14	−3.07 ± 0.21	6
EK - 22 °C - 2500 nM Ca^2+^ - 10 µM Ran	−34.88 ± 1.57	2.83 ± 0.15	8	−100.76 ± 1.83	−3.11 ± 0.13	8
EK - 22 °C - 2500 nM Ca^2+^ - 100 µM Ran	−33.72 ± 1.52	3.02 ± 0.35	5	−106.45 ± 2.29	−2.12 ± 0.08^*2^	5
EK - 34 °C - 0 nM Ca^2+^ - 0 µM Ran	−30.22 ± 1.27	3.68 ± 0.28	9	−91.02 ± 2.79	−3.18 ± 0.14	9
EK - 34 °C - 0 nM Ca^2+^ - 10 µM Ran	−36.80 ± 1.20	4.31 ± 0.38	9	−94.20 ± 3.73	−3.14 ± 0.13	9
EK - 34 °C - 0 nM Ca^2+^ - 100 µM Ran	−35.53 ± 1.18	4.11 ± 0.22	8	−105.48 ± 1.64^*1^	−2.07 ± 0.07^*2^	8
EK - 34 °C - 2500 nM Ca^2+^ - 0 µM Ran	−34.74 ± 1.48	4.06 ± 0.41	8	−96.78 ± 4.70	−3.26 ± 0.12	8
EK - 34 °C - 2500 nM Ca^2+^ - 10 µM Ran	−31.25 ± 2.04	3.92 ± 0.35	7	−95.01 ± 3.23	−2.94 ± 0.15	7
EK - 34 °C - 2500 nM Ca^2+^ - 100 µM Ran	−32.33 ± 1.64	3.39 ± 0.15	9	−105.73 ± 2.44	−2.00 ± 0.06^*2^	10

^*1^p < 0.01 vs 0 µM and 10 µM Ran of same condition.

^*2^p < 0.05 vs 0 µM and 10 µM Ran of same condition.

### E1784K availability is decreased in Ranolazine

**Figure 4:**
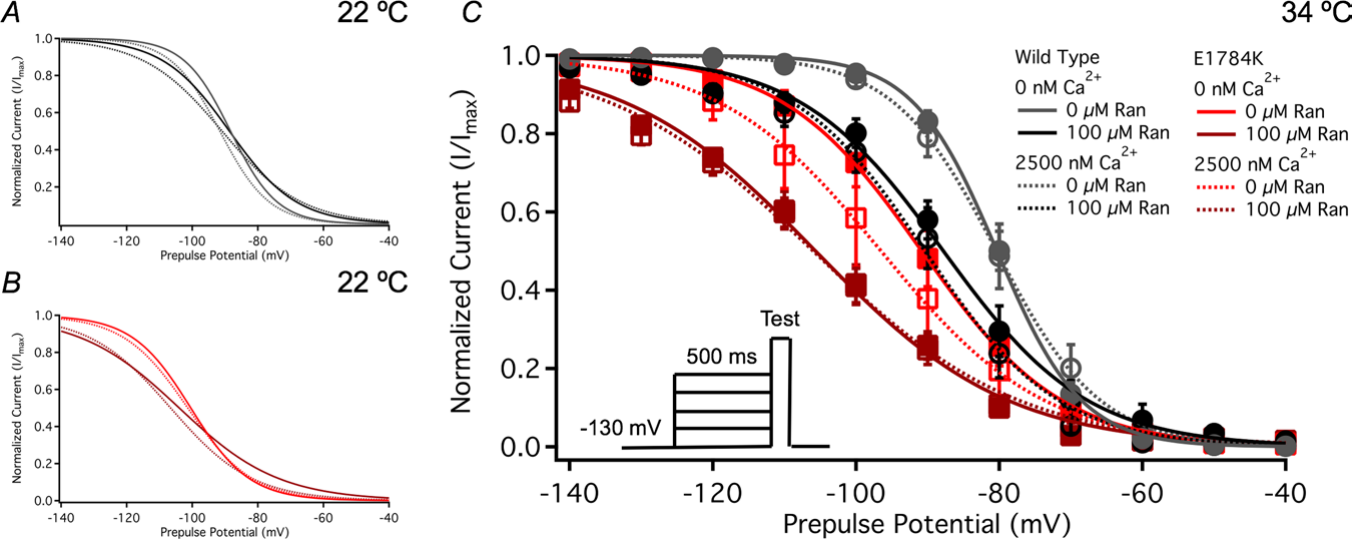
Ranolazine effects on steady-state fast inactivation. Panels A,B (22 °C) and C (34 °C) shows steady-state fast inactivation as normalized current plotted against the prepulse potential (pulse protocol shown in C *inset*).

Normalized current is plotted against membrane potential in Fig. 4. E1784K hyperpolarized (p < 0.0001) the SSFI midpoint (SSFI-V_1/2_) compared to WT. Elevated temperature depolarized (p < 0.0001) SSFI-V_1/2_ in both WT and E1784K. At 34 °C and 0 nM cytosolic calcium, SSFI-V_1/2_ in E1784K was hyperpolarized (p < 0.0001) in 100 µM Ranolazine compared to WT (Fig. 4C and Table 2). This effect was not significant at 2500 nM cytosolic calcium. Analogous to the shifts on GV-z, SSFI-z was decreased in E1784K and increased with elevated temperature (p < 0.0001). The slope was reduced in all conditions when Ranolazine was increased from 10 µM to 100 µM (p < 0.05, Table 2).

### Fast inactivation onset kinetics are not altered with Ranolazine

**Figure 5:**
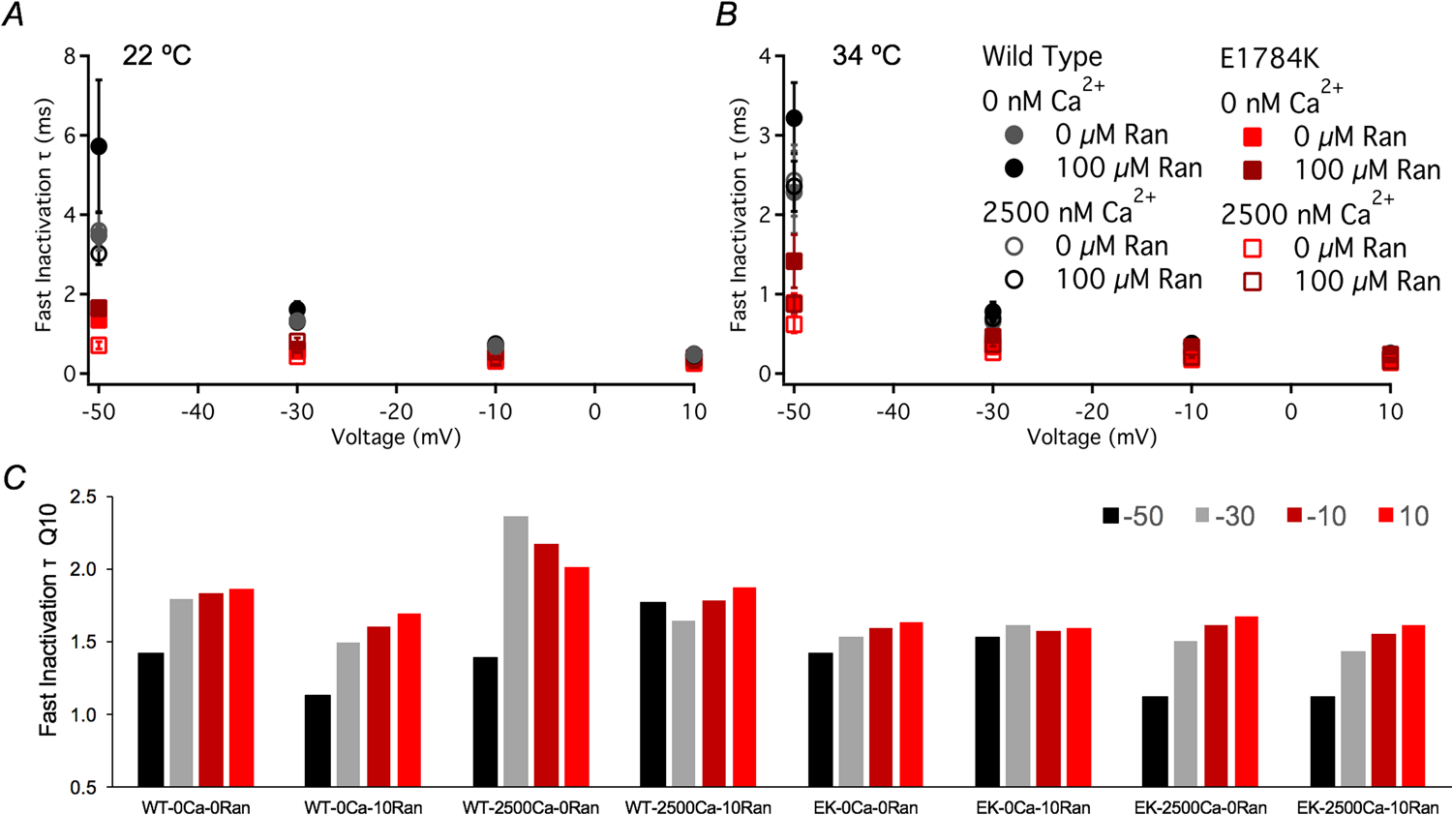
Ranolazine effects on fast inactivation onset time constants. Panels A,B show the single-exponential time constants plotted against voltage. The pulse protocol is identical to that used to measure channel conductance (refer to *Methods*). Panel C includes Q_10_ coefficient values for all conditions between −50 mV to +10 mV. Cytosolic calcium seems to modulate Ranolazine effects on WT Q_10_, as elevated cytosolic calcium heightened thermosensitivity. Cytosolic calcium made −50 mV fast inactivation onset in E1784K less thermosensitive, consistent with decoupling between CTD and Domain III-IV linker mechanism, explained in the discussion.

Fast inactivation onset kinetics at depolarized potentials (>−50 mV) were measured from τ_on_ of the mono-exponential fits. E1784K fast inactivation onset kinetics were accelerated regardless of temperature (Fig. 5A,B). Onset kinetics were accelerated (decreased τ_on_) with elevated temperature in WT compared to E1784K (p < 0.01). WT and E1784K onset kinetics were decelerated (increased τ_on_, p < 0.05) in Ranolazine as a function of voltage and cytosolic calcium at 22 °C (values reported in Table 3). These drug effects on τ_on_ were not significant at elevated temperature.

**Table 3:** Open-State Fast Inactivation Time Constant.

Condition	−50 mV τ (ms)	−30 mV τ (ms)	−10 mV τ (ms)	+10 mV τ (ms)	N
WT - 22 °C - 0 nM Ca^2+^ - 0 µM Ran	3.47 ± 0.22	1.32 ± 0.11	0.69 ± 0.07	0.48 ± 0.04	7
WT - 22 °C - 0 nM Ca^2+^ - 10 µM Ran	3.28 ± 0.45	1.13 ± 0.06	0.61 ± 0.04	0.44 ± 0.03	5
WT - 22 °C - 0 nM Ca^2+^ - 100 µM Ran	5.72 ± 1.67^*1^	1.61 ± 0.20^*2^	0.74 ± 0.06	0.46 ± 0.03	5
WT - 22 °C - 2500 nM Ca^2+^ - 0 µM Ran	3.59 ± 0.49	1.34 ± 0.12	0.69 ± 0.03	0.47 ± 0.01	8
WT - 22 °C - 2500 nM Ca^2+^ - 10 µM Ran	3.03 ± 0.13	1.09 ± 0.05	0.58 ± 0.01	0.42 ± 0.01	6
WT - 22 °C - 2500 nM Ca^2+^ - 100 µM Ran	3.02 ± 0.28	1.30 ± 0.09	0.71 ± 0.05	0.49 ± 0.04	9
WT - 34 °C - 0 nM Ca^2+^ - 0 µM Ran	2.28 ± 0.52	0.66 ± 0.07	0.34 ± 0.02	0.23 ± 0.01	5
WT - 34 °C - 0 nM Ca^2+^ - 10 µM Ran	2.82 ± 0.46	0.70 ± 0.08	0.35 ± 0.04	0.23 ± 0.02	7
WT - 34 °C - 0 nM Ca^2+^ - 100 µM Ran	3.22 ± 0.45	0.78 ± 0.12	0.38 ± 0.06	0.22 ± 0.02	5
WT - 34 °C - 2500 nM Ca^2+^ - 0 µM Ran	2.43 ± 0.45	0.48 ± 0.04	0.27 ± 0.01	0.20 ± 0.01	6
WT - 34 °C - 2500 nM Ca^2+^ - 10 µM Ran	1.53 ± 0.24	0.60 ± 0.10	0.29 ± 0.03	0.20 ± 0.02	4
WT - 34 °C - 2500 nM Ca^2+^ - 100 µM Ran	2.36 ± 0.32	0.69 ± 0.08	0.38 ± 0.04	0.25 ± 0.02	6
EK - 22 °C - 0 nM Ca^2+^ - 0 µM Ran	1.35 ± 0.14	0.57 ± 0.03	0.40 ± 0.02	0.33 ± 0.02	7
EK - 22 °C - 0 nM Ca^2+^ - 10 µM Ran	1.46 ± 0.17	0.54 ± 0.03	0.36 ± 0.02	0.30 ± 0.02	10
EK–22 °C - 0 nM Ca^2+^ - 100 µM Ran	1.65 ± 0.16	0.61 ± 0.03	0.37 ± 0.01	0.29 ± 0.01	6
EK - 22 °C - 2500 nM Ca^2+^ - 0 µM Ran	0.71 ± 0.09	0.44 ± 0.02	0.32 ± 0.01	0.26 ± 0.01	6
EK - 22 °C - 2500 nM Ca^2+^ - 10 µM Ran	1.23 ± 0.09	0.54 ± 0.03	0.37 ± 0.01	0.30 ± 0.01	7
EK - 22 °C - 2500 nM Ca^2+^ - 100 µM Ran	1.65 ± 0.13	0.81 ± 0.09	0.54 ± 0.06^*3^	0.40 ± 0.03^*4^	5
EK - 34 °C - 0 nM Ca^2+^ - 0 µM Ran	0.89 ± 0.12	0.34 ± 0.02	0.23 ± 0.02	0.18 ± 0.01	9
EK - 34 °C - 0 nM Ca^2+^ - 10 µM Ran	0.88 ± 0.11	0.30 ± 0.02	0.21 ± 0.01	0.17 ± 0.01	9
EK - 34 °C - 0 nM Ca^2+^ - 100 µM Ran	1.41 ± 0.34	0.47 ± 0.09	0.34 ± 0.07	0.24 ± 0.05	8
EK - 34 °C - 2500 nM Ca^2+^ - 0 µM Ran	0.62 ± 0.11	0.27 ± 0.02	0.18 ± 0.01	0.14 ± 0.01	9
EK - 34 °C - 2500 nM Ca^2+^ - 10 µM Ran	1.08 ± 0.07	0.35 ± 0.05	0.22 ± 0.02	0.17 ± 0.01	6
EK - 34 °C - 2500 nM Ca^2+^ - 100 µM Ran	0.88 ± 0.11	0.37 ± 0.02	0.21 ± 0.01	0.16 ± 0.01	8

*^1^p < 0.05 vs 0 µM and 10 µM Ran of same condition.

*^2^p < 0.05 vs 10 µM Ran of same condition.

*^3^p < 0.01 vs 0 µM Ran of same condition.

*^4^p < 0 ± 0.05 vs 0 µM Ran of same condition.

Figure 5C shows the Q_10_ values at 0 µM and 10 µM Ranolazine for all conditions. We observed high variability in the temperature coefficient at −50 mV compared to other voltages. At −50 mV, both Ranolazine and cytosolic calcium mutually affect thermosensitivity in WT: Q_10_ decreased at 0 nM cytosolic calcium and increased at 2500 nM cytosolic calcium in 10 µM Ranolazine. At more depolarized voltages than −50 mV, subtle alterations occurred in Q_10_ (Fig. 5C). E1784K Q_10_ was not sensitive to Ranolazine. However, E1784K thermosensitivity was dampened in cytosolic calcium at −50 mV compared to other voltages and to WT.

### Ranolazine does not suppress thermosensitive late INa in E1784K with elevated cytosolic calcium

**Figure 6:**
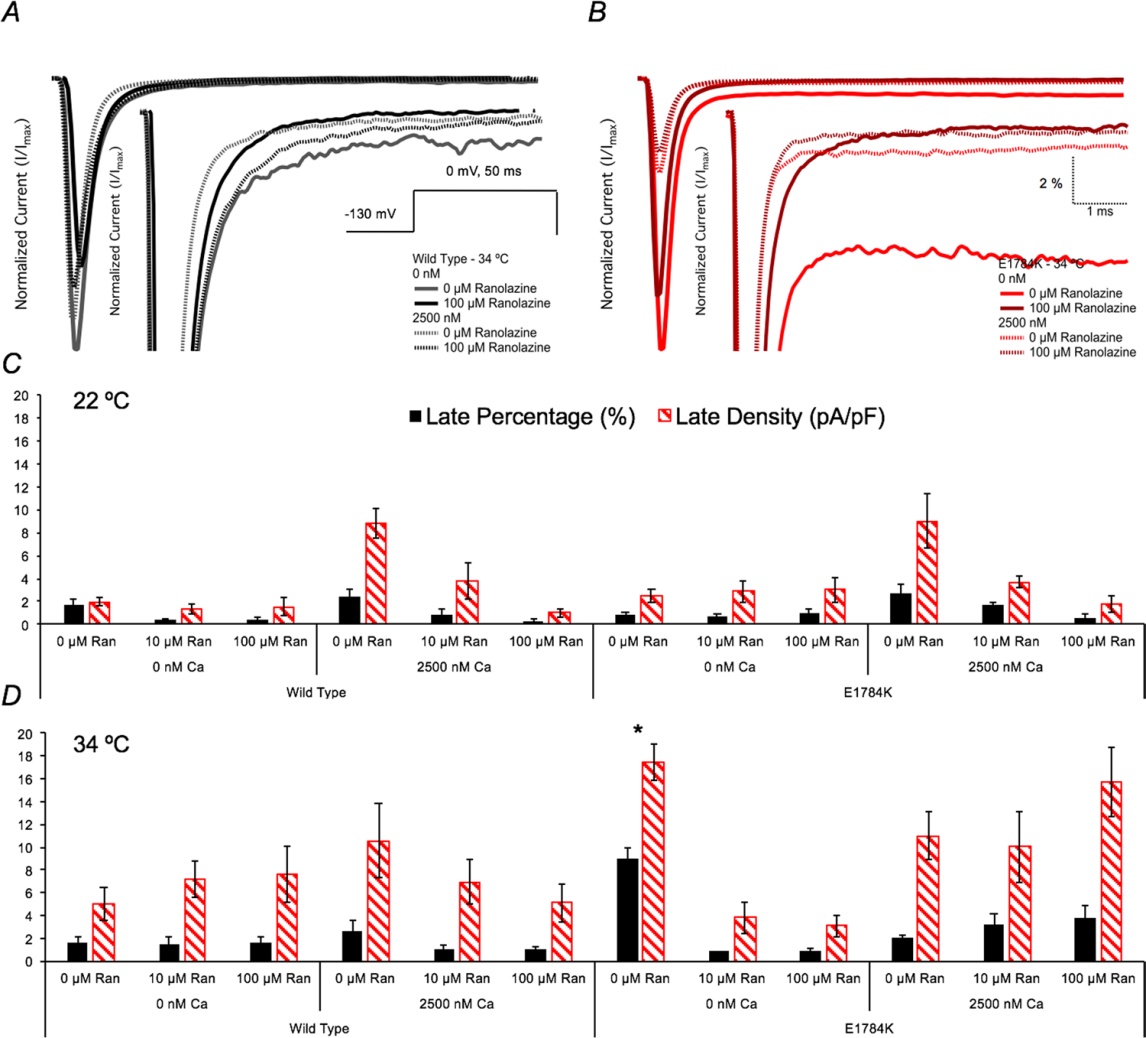
Ranolazine effects on late I_Na_. Panels A,B show normalized current traces, with emphasis on the non-inactivating, late I_Na_, at 34 °C. The normalized late I_Na_ in E1784K is enhanced drastically by elevated temperature. Cytosolic calcium suppresses the E1784K normalized late I_Na_ (addressed in discussions). Ranolazine suppresses late I_Na_ only at 0 nM compared to 2500 nM cytosolic calcium. Panels C,D show the late I_Na_ percent and density bar graphs for all conditions at 22 °C and 34 °C, respectively.

Representative normalized late I_Na_ current traces are shown in Fig. 6A,B at 0 µM and 100 µM Ranolazine (only 34 °C shown). Late I_Na_ percent and density are shown in Fig. 6C,D as bar graphs. Late I_Na_ percent and density in E1784K increased (p < 0.01) by 11-fold and 7-fold, respectively, with elevated temperature at 0 nM cytosolic calcium (Fig. 6D and Table 4). This increase in late I_Na_ was almost fully attenuated in 10 µM Ranolazine. Late I_Na_ percent decreased in elevated cytosolic calcium (p < 0.01) but there was no effect on late I_Na_ density in E1784K. Late I_Na_ percent and density in E1784K were not suppressed with Ranolazine at 2500 nM cytosolic calcium (Fig. 6D and Table 4).

**Table 4:** Late I_Na_ Current Density and Percentage.

Condition	Late I_Na_ Density (pA/pF)	N	Late Percent (%)	N
WT - 22 °C - 0 nM Ca^2+^ - 0 µM Ran	1.95 ± 0.34	9	1.71 ± 0.54	9
WT - 22 °C - 0 nM Ca^2+^ - 10 µM Ran	1.35 ± 0.44	8	0.35 ± 0.12	8
WT - 22 °C - 0 nM Ca^2+^ - 100 µM Ran	1.55 ± 0.79	6	0.45 ± 0.20	6
WT - 22 °C - 2500 nM Ca^2+^ - 0 µM Ran	8.83 ± 1.33	8	2.41 ± 0.72	6
WT - 22 °C - 2500 nM Ca^2+^ - 10 µM Ran	3.78 ± 1.64	8	0.80 ± 0.48	7
WT - 22 °C - 2500 nM Ca^2+^ - 100 µM Ran	1.01 ± 0.38	7	0.30 ± 0.12	6
WT - 34 °C - 0 nM Ca^2+^ - 0 µM Ran	5.04 ± 1.42	7	1.57 ± 0.52	9
WT - 34 °C - 0 nM Ca^2+^ - 10 µM Ran	7.21 ± 1.61	8	1.54 ± 0.54	8
WT - 34 °C - 0 nM Ca^2+^ - 100 µM Ran	7.66 ± 2.47	9	1.67 ± 0.42	9
WT - 34 °C - 2500 nM Ca^2+^ - 0 µM Ran	10.57 ± 3.2	7	2.71 ± 0.90	9
WT - 34 °C - 2500 nM Ca^2+^ - 10 µM Ran	6.95 ± 1.97	8	1.09 ± 0.26	8
WT - 34 °C - 2500 nM Ca^2+^ - 100 µM Ran	5.11 ± 1.67	4	0.98 ± 0.25	5
EK - 22 °C - 0 nM Ca^2+^ - 0 µM Ran	2.51 ± 0.63	9	0.85 ± 0.20	9
EK - 22 °C - 0 nM Ca^2+^ - 10 µM Ran	2.86 ± 0.88	9	0.68 ± 0.21	8
EK - 22 °C - 0 nM Ca^2+^ - 100 µM Ran	3.01 ± 1.08	10	1.02 ± 0.39	8
EK - 22 °C - 2500 nM Ca^2+^ - 0 µM Ran	9.02 ± 2.31	5	2.64 ± 0.81	4
EK - 22 °C - 2500 nM Ca^2+^ - 10 µM Ran	3.68 ± 0.51	9	1.65 ± 0.30	9
EK - 22 °C - 2500 nM Ca^2+^ - 100 µM Ran	1.78 ± 0.65	8	0.61 ± 0.30	6
EK - 34 °C - 0 nM Ca^2+^ - 0 µM Ran	17.47 ± 1.54^*1^	6	8.97 ± 1.02^*1^	6
EK - 34 °C - 0 nM Ca^2+^ - 10 µM Ran	3.84 ± 1.39	4	0.86 ± 0.04	4
EK - 34 °C - 0 nM Ca^2+^ - 100 µM Ran	3.09 ± 0.96	6	0.89 ± 0.22	6
EK - 34 °C - 2500 nM Ca^2+^ - 0 µM Ran	11.01 ± 2.12	7	2.00 ± 0.34	9
EK - 34 °C - 2500 nM Ca^2+^ - 10 µM Ran	10.05 ± 3.08	5	3.28 ± 0.93	6
EK - 34 °C - 2500 nM Ca^2+^ - 100 µM Ran	15.73 ± 6.24	5	3.76 ± 1.10	4

^*1^p < 0.01 vs 10 µM and 100 µM Ran of same condition.

### Ranolazine does not enhance UDI in E1784K with elevated cytosolic calcium

**Figure 7:**
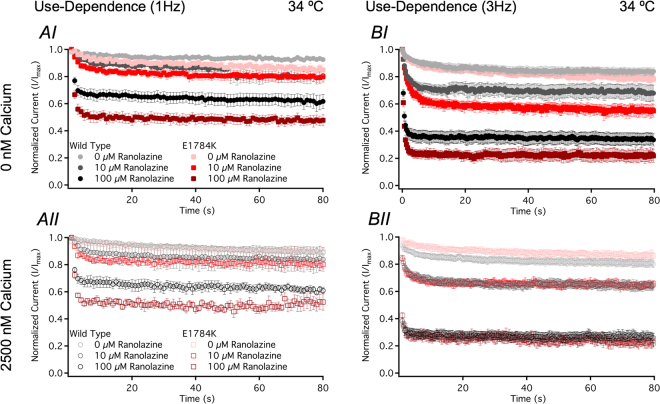
Ranolazine effects on use-dependence. Panels AI-II and Panels BI-II show normalized current versus time measuring UDI at 1 Hz and 3 Hz, respectively. Insets are excluded for visual clarity (refer to methods for pulse protocols). All three drug concentrations are included in the figures to show 10 µM Ranolazine effect on UDI (3 Hz) in E1784K at 0 nM compared to 2500 nM.

Sustained or repetitive depolarizations induce slow inactivation in Na_V_1.5, which was indirectly measured by the use-dependent inactivation (UDI) protocols described in the methods. We were unable to measure fast inactivation recovery kinetics in E1784K which may be decelerated by Ranolazine and contribute to UDI^54^. Use-dependence was measured at 1 Hz and 3 Hz, mimicking resting heart rate (60 bpm) and tachycardia (180 bpm), respectively. Normalized current plotted against time for UDI measured at 1 Hz and 3 Hz are shown in Fig. 7 (only 34 °C shown).

UDI plateau (y_0_) was greater (p = 0.0430) at elevated temperature at both 1 Hz and 3 Hz, but the shift was larger in E1784K at 1 Hz (Table 5). y_0_ decreased to different levels in Ranolazine (reported in Table 5). At high UDI frequencies, E1784K y_0_ decreased in Ranolazine at 34 °C compared to WT (Fig. 7 shows only 34 °C, Table 5). Our statistical results suggest that the drug effects on UDI (3 Hz) in E1784K are limited in elevated cytosolic calcium (Table 5).

**Table 5:** Use-Dependence (1 Hz and 3 Hz).

Condition	1 Hz–y0	1 Hz–τ1 (s)	1 Hz–τ2 (s)	N	3 Hz–y0	3 Hz–τ1 (s)	3 Hz–τ2 (s)	N
WT - 22 °C - 0 nM Ca^2+^ - 0 µM Ran	0.84 ± 0.03	3.13 ± 0.88	47.88 ± 9.44	6	0.63 ± 0.04	2.03 ± 0.41	40.53 ± 5.42	7
WT - 22 °C - 0 nM Ca^2+^ - 10 µM Ran	0.77 ± 0.01	2.96 ± 0.52	18.55 ± 4.50	7	0.52 ± 0.02	3.39 ± 0.62	23.00 ± 7.54	7
WT - 22 °C - 0 nM Ca^2+^ - 100 µM Ran	0.44 ± 0.02^*1^	2.19 ± 0.44	9.70 ± 3.31	5	0.15 ± 0.02^*1^	0.92 ± 0.18	7.02 ± 4.44	4
WT - 22 °C - 2500 nM Ca^2+^ - 0 µM Ran	0.81 ± 0.02	8.22 ± 2.84	114.32 ± 22.42	7	0.66 ± 0.03	1.83 ± 0.23	36.4 ± 7.98	7
WT - 22 °C - 2500 nM Ca^2+^ - 10 µM Ran	0.78 ± 0.01	6.93 ± 0.94	31.93 ± 10.85	6	0.52 ± 0.02	1.19 ± 0.19	8.57 ± 0.42	5
WT - 22 °C - 2500 nM Ca^2+^ - 100 µM Ran	0.35 ± 0.03^*1^	1.67 ± 0.12	34.32 ± 22.87	7	0.20 ± 0.03^*1^	0.69 ± 0.32	14.56 ± 13.09	3
WT - 34 °C - 0 nM Ca^2+^ - 0 µM Ran	0.92 ± 0.01	4.77 ± 3.14	39.42 ± 20.36	4	0.84 ± 0.03	2.75 ± 1.00	17.73 ± 9.63	5
WT - 34 °C - 0 nM Ca^2+^ - 10 µM Ran	0.76 ± 0.05	5.41 ± 3.35	62.60 ± 20.25	5	0.65 ± 0.06	0.98 ± 0.10	16.65 ± 6.29	6
WT - 34 °C - 0 nM Ca^2+^ - 100 µM Ran	0.60 ± 0.06^*2^	0.66 ± 0.10	43.51 ± 18.93	8	0.33 ± 0.05^*1^	0.39 ± 0.08	13.99 ± 8.03	8
WT - 34 °C - 2500 nM Ca^2+^ - 0 µM Ran	0.88 ± 0.03	30.10 ± 6.41^*3^	59.96 ± 37.08	4	0.79 ± 0.04	1.46 ± 0.17	38.85 ± 12.19	8
WT - 34 °C - 2500 nM Ca^2+^ - 10 µM Ran	0.82 ± 0.04	2.83 ± 1.02	67.05 ± 23.05	5	0.60 ± 0.03^*4^	1.02 ± 0.16	17.28 ± 6.89	7
WT - 34 °C - 2500 nM Ca^2+^ - 100 µM Ran	0.64 ± 0.02	0.44 ± 0.16	8.44 ± 2.77	5	0.27 ± 0.03^*1^	0.41 ± 0.06	17.80 ± 9.98	5
EK - 22 °C - 0 nM Ca^2+^ - 0 µM Ran	0.69 ± 0.06	3.37 ± 0.64	69.20 ± 25.09	6	0.79 ± 0.01	3.08 ± 1.61	32.65 ± 20.62	4
EK - 22 °C - 0 nM Ca^2+^ - 10 µM Ran	0.57 ± 0.05	3.25 ± 0.63	64.71 ± 37.46	7	0.47 ± 0.02^*4^	2.40 ± 0.37	16.28 ± 3.57	6
EK - 22 °C - 0 nM Ca^2+^ - 100 µM Ran	0.27 ± 0.05^*1^	1.87 ± 0.25	74.43 ± 28.56	8	0.14 ± 0.03^*1^	1.00 ± 0.34	1.29 ± 0.19	4
EK - 22 °C - 2500 nM Ca^2+^ - 0 µM Ran	0.73 ± 0.02	1.99 ± 1.02	31.37 ± 6.74	4	0.69 ± 0.06	3.59 ± 1.84	8.20 ± 3.73	4
EK - 22 °C - 2500 nM Ca^2+^ - 10 µM Ran	0.59 ± 0.06	7.17 ± 1.35	75.52 ± 20.26	5	0.47 ± 0.04	3.38 ± 0.43	13.21 ± 1.51	5
EK - 22 °C - 2500 nM Ca^2+^ - 100 µM Ran	0.24 ± 0.04^*1^	2.68 ± 1.13	70.86 ± 29.29	3	0.20 ± 0.06^*2^	0.82 ± 0.29	1.94 ± 0.31	3
EK - 34 °C - 0 nM Ca^2+^ - 0 µM Ran	0.82 ± 0.04	6.76 ± 1.43	68.66 ± 25.39	8	0.77 ± 0.03	2.66 ± 0.45	49.11 ± 13.27	9
EK - 34 °C - 0 nM Ca^2+^ - 10 µM Ran	0.77 ± 0.06	2.40 ± 0.55	78.22 ± 35.32	7	0.55 ± 0.03^*4^	1.63 ± 0.32	27.16 ± 15.43	5
EK - 34 °C - 0 nM Ca^2+^ - 100 µM Ran	0.47 ± 0.04^*1^	0.77 ± 0.07	19.07 ± 9.49	6	0.21 ± 0.05^*1^	0.40 ± 0.04	24.08 ± 18.78	5
EK - 34 °C - 2500 nM Ca^2+^ - 0 µM Ran	0.87 ± 0.03	15.05 ± 3.35^*3^	65.60 ± 19.19	5	0.74 ± 0.08	2.50 ± 0.65	30.82 ± 2.43	6
EK - 34 °C - 2500 nM Ca^2+^ - 10 µM Ran	0.77 ± 0.04	1.77 ± 0.48	48.78 ± 22.76	5	0.63 ± 0.03	1.13 ± 0.12	6.41 ± 3.13	6
EK - 34 °C - 2500 nM Ca^2+^ - 100 µM Ran	0.46 ± 0.05^*1^	1.21 ± 0.20	38.24 ± 19.24	5	0.26 ± 0.03^*1^	0.54 ± 0.14	6.89 ± 4.62	5

^*1^p < 0.0001 vs 0 µM and 10 µM Ran of same condition.

^*2^p < 0.0001 vs 0 µM Ran of same condition.

^*3^p < 0.0001 vs 10 µM and 100 µM Ran of same condition.

^*4^p < 0.0001 vs 0 µM and 100 µM Ran of same condition.

UDI onset kinetics were accelerated in E1784K and elevated temperature (p < 0.05), measured by τ_1_, at 1 Hz and 3 Hz. Onset kinetics decelerated in elevated cytosolic calcium (p < 0.0001) at 1 Hz predominately in WT compared to E1784K. WT and E1784K τ_1_ was decreased (p < 0.0001) in Ranolazine at 2500 nM cytosolic calcium and 34 °C (Table 5). τ_1_ at 3 Hz was unaffected in Ranolazine. τ_2_ was unaffected at 1 Hz and 3 Hz in all experimental conditions.

### E1784K-induced alternans is exacerbated with Ranolazine

**Figure 8:**
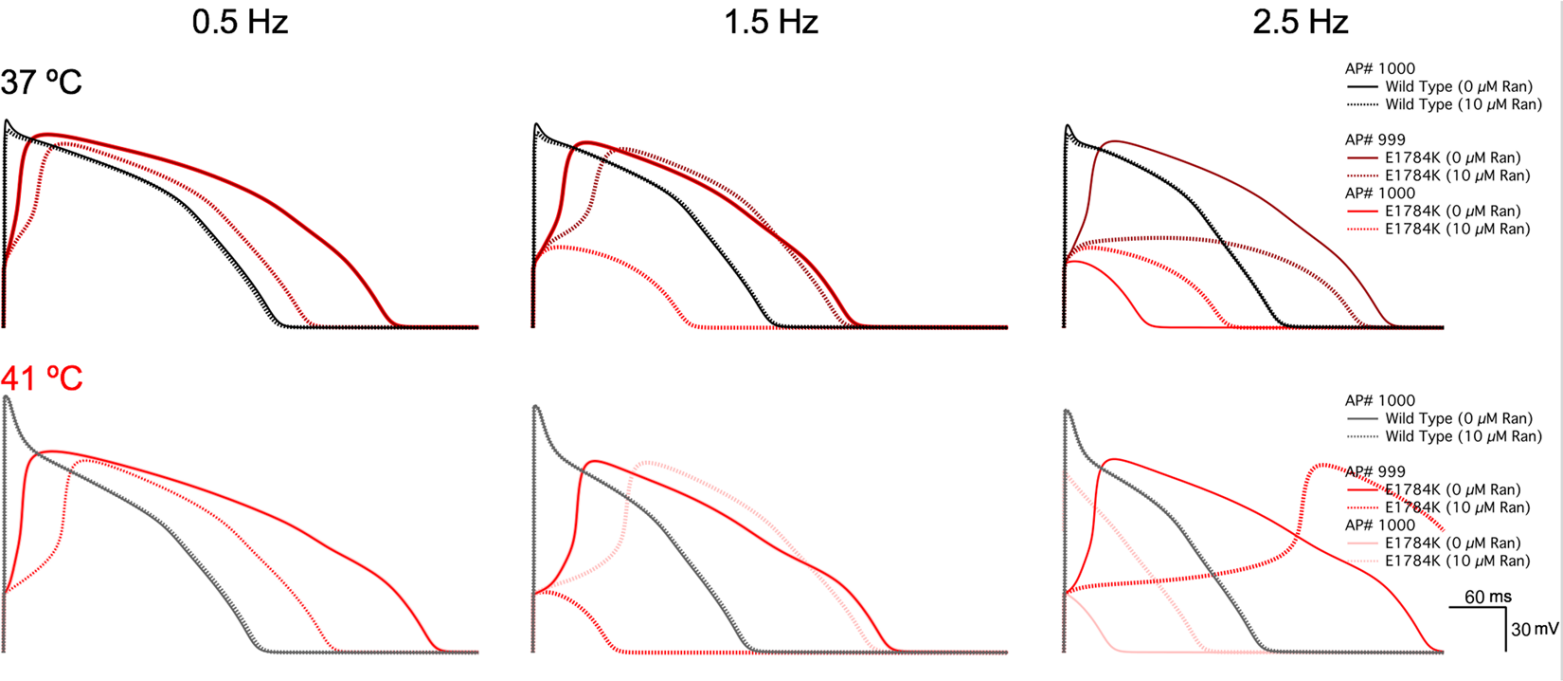
Endocardial action potential simulations. AP simulations are plotted against time (*inset* is shown in bottom right corner) at 37 °C and 41 °C. The last two AP beats were plotted in E1784K to show alternans-induction. Simulations only included therapeutic concentrations of Ranolazine (10 µM).

Biophysical data were extrapolated to physiological (37 °C) and febrile (41 °C) temperatures using a Q_10_ coefficient (Equation 6). Extrapolations were independently supported using Arrhenius relationships (Equation 7 and Table 6). Action potential (AP) traces are shown in Fig. 8 for simulations conducted in endocardial cells at three frequencies: 0.5 Hz (bradycardia), 1.5 Hz (sinus rhythm), and 2.5 Hz (tachycardia). At febrile temperature, simulated APs show accelerated depolarizations and repolarizations in WT compared to E1784K.

**Table 6:** Values at 37 °C and 41 °C based on Q_10_ and Arrhenius Calculations.

**Condition**	**37 °C** **(Q**_**10**_ **)**	**41 °C** **(Q**_**10**_ **)**	**37 °C** **(Arrhenius)**	**41 °C** **(Arrhenius)**
**Peak GV Density (nS/pF)**				
WT - 0 nM Ca^2+^ - 0 µM Ran	16179.18	55592.62	8124.68	10881.05
WT - 0 nM Ca^2+^ - 10 µM Ran	22678.58	99329.99	9107.70	11251.36
WT - 2500 nM Ca^2+^ - 0 µM Ran	27586.50	131656.89	9377.01	11024.82
WT - 2500 nM Ca^2+^ - 10 µM Ran	15043.82	48489.81	6439.00	6969.03
EK - 0 nM Ca^2+^ - 0 µM Ran	3575.20	3527.30	3569.09	3525.15
EK - 0 nM Ca^2+^ - 10 µM Ran	12400.15	35482.80	5731.14	6055.55
EK - 2500 nM Ca^2+^ - 0 µM Ran	14940.82	51538.73	6366.24	7251.15
EK - 2500 nM Ca^2+^ - 10 µM Ran	2860.49	2527.87	2816.21	2513.95
**Peak I**_**Na**_ **Density (pA/pF)**				
WT - 0 nM Ca^2+^ - 0 µM Ran	446.63	597.28	463.00	604.67
WT - 0 nM Ca^2+^ - 10 µM Ran	564.39	706.75	580.56	713.78
WT - 2500 nM Ca^2+^ - 0 µM Ran	623.14	754.26	637.95	760.26
WT - 2500 nM Ca^2+^ - 10 µM Ran	521.76	633.36	534.72	638.92
EK - 0 nM Ca^2+^ - 0 µM Ran	270.93	288.18	272.97	288.90
EK - 0 nM Ca^2+^ - 10 µM Ran	339.21	361.89	341.86	362.80
EK - 2500 nM Ca^2+^ - 0 µM Ran	430.22	549.75	443.92	556.03
EK - 2500 nM Ca^2+^ - 10 µM Ran	222.38	215.51	221.51	215.22
**Late I**_**Na**_ **Density (pA/pF)**				
WT - 0 nM Ca^2+^ - 0 µM Ran	6.48	8.89	6.74	9.01
WT - 0 nM Ca^2+^ - 10 µM Ran	11.26	19.76	12.09	20.26
WT - 2500 nM Ca^2+^ - 0 µM Ran	11.08	11.77	11.17	11.80
WT–2500 nM Ca^2+^ - 10 µM Ran	8.17	10.02	8.38	10.11
EK - 0 nM Ca^2+^ - 0 µM Ran	29.25	56.02	31.75	57.68
EK - 0 nM Ca^2+^ - 10 µM Ran	4.15	4.58	4.20	4.60
EK - 2500 nM Ca^2+^ - 0 µM Ran	11.61	12.41	11.71	12.45
EK - 2500 nM Ca^2+^ - 10 µM Ran	13.12	18.37	13.69	18.65
**Late Percent (%)**				
WT - 0 nM Ca^2+^ - 0 µM Ran	1.54	1.50	1.53	1.49
WT - 0 nM Ca^2+^ - 10 µM Ran	2.27	3.71	2.41	3.79
WT - 2500 nM Ca^2+^ - 0 µM Ran	2.80	2.91	2.81	2.92
WT - 2500 nM Ca^2+^ - 10 µM Ran	1.18	1.30	1.19	1.31
EK - 0 nM Ca^2+^ - 0 µM Ran	11.64	16.17	12.13	16.41
EKv0 nM Ca^2+^ - 10 µM Ran	0.92	1.00	0.93	1.00
EK - 2500 nM Ca^2+^ - 0 µM Ran	1.86	1.69	1.84	1.69
EK - 2500 nM Ca^2+^ - 10 µM Ran	3.94	4.96	4.05	5.01
**GV-V**_**1/2**_ **(mV)**				
WT - 0 nM Ca^2+^ - 0 µM Ran	−44.27	−45.34	−44.40	−45.39
WT - 0 nM Ca^2+^ - 10 µM Ran	−36.12	−33.96	−35.84	−33.87
WT - 2500 nM Ca^2+^ - 0 µM Ran	−40.20	−39.68	−40.13	−39.66
WT - 2500 nM Ca^2+^ - 10 µM Ran	−36.50	−34.66	−36.26	−34.58
EK - 0 nM Ca^2+^ - 0 µM Ran	−29.04	−27.61	−28.86	−27.56
EK - 0 nM Ca^2+^ - 10 µM Ran	−37.06	−37.39	−37.10	−37.40
EK - 2500 nM Ca^2+^ - 0 µM Ran	−33.62	−32.25	−33.44	−32.19
EK - 2500 nM Ca^2+^ - 10 µM Ran	−30.35	−29.25	−30.21	−29.21
**GV-z**				
WT - 0 nM Ca^2+^ - 0 µM Ran	6.79	7.93	6.92	7.99
WT - 0 nM Ca^2+^ - 10 µM Ran	4.84	4.71	4.82	4.71
WT - 2500 nM Ca^2+^ - 0 µM Ran	6.59	7.49	6.70	7.53
WT - 2500 nM Ca^2+^ - 10 µM Ran	5.55	6.11	5.62	6.13
EK - 0 nM Ca^2+^ - 0 µM Ran	3.89	4.17	3.93	4.19
EK - 0 nM Ca^2+^ - 10 µM Ran	4.68	5.21	4.75	5.23
EK - 2500 nM Ca^2+^ - 0 µM Ran	4.36	4.78	4.41	4.80
EK - 2500 nM Ca^2+^ - 10 µM Ran	4.28	4.78	4.34	4.80
**SSFI-V**_**1/2**_ **(mV)**				
WT - 0 nM Ca^2+^ - 0 µM Ran	−78.24	−75.70	−77.91	−75.59
WT - 0 nM Ca^2+^ - 10 µM Ran	−81.49	−80.21	−81.32	−80.15
WT - 2500 nM Ca^2+^ - 0 µM Ran	−77.46	−74.07	−77.02	−73.92
WT - 2500 nM Ca^2+^ - 10 µM Ran	−81.11	−78.58	−80.79	−78.47
EK - 0 nM Ca^2+^ - 0 µM Ran	−88.67	−85.79	−88.30	−85.67
EK - 0 nM Ca^2+^–10 µM Ran	−92.87	−91.23	−92.66	−91.15
EK - 2500 nM Ca^2+^ - 0 µM Ran	−95.62	−94.16	−95.43	−94.10
EK - 2500 nM Ca^2+^ - 10 µM Ran	−93.54	−91.72	−93.31	−91.64
**SSFI**-**z**				
WT - 0 nM Ca^2+^ - 0 µM Ran	−4.95	−5.26	−4.99	−5.27
WT - 0 nM Ca^2+^ - 10 µM Ran	−4.02	−4.04	−4.02	−4.04
WT - 2500 nM Ca^2+^ - 0 µM Ran	−4.41	−4.63	−4.44	−4.63
WT - 2500 nM Ca^2+^ - 10 µM Ran	−4.02	−4.05	−4.03	−4.05
EK - 0 nM Ca^2+^ - 0 µM Ran	−3.24	−3.31	−3.25	−3.32
EK - 0 nM Ca^2+^ - 10 µM Ran	−3.21	−3.30	−3.22	−3.31
EK - 2500 nM Ca^2+^ - 0 µM Ran	−3.31	−3.38	−3.32	−3.38
EK - 2500 nM Ca^2+^ - 10 µM Ran	−2.90	−2.85	−2.89	−2.84
−**50mV FI τ (ms)**				
WT - 0 nM Ca^2+^ - 0 µM Ran	2.04	1.77	2.00	1.76
WT - 0 nM Ca^2+^ - 10 µM Ran	2.71	2.58	2.69	2.57
WT - 2500 nM Ca^2+^ - 0 µM Ran	2.19	1.92	2.15	1.91
WT - 2500 nM Ca^2+^ - 10 µM Ran	1.27	1.01	1.24	1.00
EK - 0 nM Ca^2+^ - 0 µM Ran	0.80	0.69	0.78	0.69
EK - 0 nM Ca^2+^ - 10 µM Ran	0.77	0.65	0.75	0.64
EK - 2500 nM Ca^2+^ - - 0 µM Ran	0.60	0.57	0.59	0.57
EK - 2500 nM Ca^2+^ - 10 µM Ran	1.04	0.99	1.03	0.99
−**30mV FI τ (ms)**				
WT–0 nM Ca^2+^ - 0 µM Ran	0.54	0.43	0.53	0.43
WT - 0 nM Ca^2+^–10 µM Ran	0.61	0.52	0.60	0.52
WT - 2500 nM Ca^2+^ - 0 µM Ran	0.36	0.26	0.35	0.25
WT - 2500 nM Ca^2+^ - 10 µM Ran	0.51	0.42	0.50	0.42
EK - 0 nM Ca^2+^ - 0 µM Ran	0.30	0.25	0.29	0.25
EK - 0 nM Ca^2+^ - 10 µM Ran	0.26	0.21	0.25	0.21
EK - 2500 nM Ca^2+^ - 0 µM Ran	0.23	0.20	0.23	0.20
EK - 2500 nM Ca^2+^ - 10 µM Ran	0.31	0.27	0.31	0.27
−**10mV FI τ (ms)**				
WT - 0 nM Ca^2+^ - 0 µM Ran	0.28	0.22	0.27	0.21
WT - 0 nM Ca^2+^ - 10 µM Ran	0.30	0.25	0.29	0.25
WT - 2500 nM Ca^2+^ - 0 µM Ran	0.21	0.16	0.20	0.15
WT–2500 nM Ca^2+^ - 10 µM Ran	0.24	0.19	0.23	0.19
EK - 0 nM Ca^2+^ - 0 µM Ran	0.20	0.16	0.19	0.16
EK - 0 nM Ca^2+^ - 10 µM Ran	0.18	0.15	0.18	0.15
EK - 2500 nM Ca^2+^ - 0 µM Ran	0.15	0.13	0.15	0.13
EK - 2500 nM Ca^2+^ - 10 µM Ran	0.19	0.16	0.18	0.16
** +10 mV FI τ (ms)**				
WT - 0 nM Ca^2+^ - 0 µM Ran	0.19	0.14	0.18	0.14
WT - 0 nM Ca^2+^ - 10 µM Ran	0.20	0.16	0.19	0.16
WT - 2500 nM Ca^2+^ - 0 µM Ran	0.16	0.12	0.16	0.12
WT - 2500 nM Ca^2+^ - 10 µM Ran	0.16	0.13	0.16	0.12
EK - 0 nM Ca^2+^ - 0 µM Ran	0.16	0.13	0.15	0.13
EK - 0 nM Ca^2+^ - 10 µM Ran	0.15	0.12	0.14	0.12
EK - 2500 nM Ca^2+^ - 0 µM Ran	0.12	0.10	0.12	0.10
EK - 2500 nM Ca^2+^ - 10 µM Ran	0.15	0.12	0.14	0.12
**1 Hz-y0**				
WT - 0 nM Ca^2+^ - 0 µM Ran	0.94	0.97	0.94	0.97
WT - 0 nM Ca^2+^ - 10 µM Ran	0.76	0.76	0.76	0.76
WT - 2500 nM Ca^2+^ - 0 µM Ran	0.90	0.93	0.90	0.93
WT - 2500 nM Ca^2+^ - 10 µM Ran	0.83	0.85	0.84	0.85
EK - 0 nM Ca^2+^ - 0 µM Ran	0.86	0.92	0.87	0.92
EK - 0 nM Ca^2+^ - 10 µM Ran	0.83	0.91	0.84	0.92
EK - 2500 nM Ca^2+^ - 0 µM Ran	0.92	0.97	0.92	0.97
EK - 2500 nM Ca^2+^ - 10 µM Ran	0.83	0.90	0.84	0.91
**1 Hz-τ1 (s)**				
WT - 0 nM Ca^2+^ - 0 µM Ran	5.34	6.15	5.43	6.18
WT - 0 nM Ca^2+^ - 10 µM Ran	6.34	7.76	6.51	7.83
WT - 2500 nM Ca^2+^ - 0 µM Ran	42.49	65.65	44.88	66.93
WT - 2500 nM Ca^2+^ - 10 µM Ran	2.23	1.65	2.15	1.63
EK - 0 nM Ca^2+^ - 0 µM Ran	8.14	10.28	8.38	10.38
EK - 0 nM Ca^2+^ - 10 µM Ran	2.22	2.01	2.19	2.00
EK - 2500 nM Ca^2+^ - 0 µM Ran	25.75	50.72	28.04	52.25
EK - 2500 nM Ca^2+^ - 10 µM Ran	1.22	0.77	1.15	0.75
**1 Hz-τ2 (s)**				
WT - 0 nM Ca^2+^ - 0 µM Ran	37.43	35.07	37.13	34.97
WT - 0 nM Ca^2+^ - 10 µM Ran	86.46	129.96	91.00	132.31
WT - 2500 nM Ca^2+^ - 0 µM Ran	50.52	40.70	49.16	40.30
WT - 2500 nM Ca^2+^ - 10 µM Ran	81.65	104.69	84.25	105.85
EK - 0 nM Ca^2+^ - 0 µM Ran	68.52	68.34	68.50	68.33
EK - 0 nM Ca^2+^ - 10 µM Ran	82.26	87.65	82.91	87.89
EK - 2500 nM Ca^2+^ - 0 µM Ran	79.80	102.18	82.29	103.26
EK - 2500 nM Ca^2+^ - 10 µM Ran	43.43	37.52	42.64	37.27
**3 Hz-y0**				
WT - 0 nM Ca^2+^ - 0 µM Ran	0.90	0.99	0.91	1.00
WT - 0 nM Ca^2+^ - 10 µM Ran	0.69	0.75	0.70	0.75
WT - 2500 nM Ca^2+^ - 0 µM Ran	0.83	0.88	0.84	0.88
WT - 2500 nM Ca^2+^ - 10 µM Ran	0.63	0.65	0.63	0.66
EK - 0 nM Ca^2+^ - 0 µM Ran	0.79	0.82	0.79	0.83
EK - 0 nM Ca^2+^ - 10 µM Ran	0.57	0.60	0.57	0.60
EK - 2500 nM Ca^2+^ - 0 µM Ran	0.76	0.78	0.76	0.78
EK - 2500 nM Ca^2+^ - 10 µM Ran	0.68	0.76	0.69	0.76
**3 Hz-τ1 (s)**				
WT - 0 nM Ca^2+^ - 0 µM Ran	2.97	3.29	3.01	3.30
WT - 0 nM Ca^2+^ - 10 µM Ran	0.70	0.46	0.67	0.46
WT - 2500 nM Ca^2+^ - 0 µM Ran	1.38	1.28	1.36	1.27
WT - 2500 nM Ca^2+^ - 10 µM Ran	0.98	0.93	0.97	0.92
EK - 0 nM Ca^2+^ - 0 µM Ran	3.07	3.69	3.14	3.72
EK - 0 nM Ca^2+^ - 10 µM Ran	1.47	1.29	1.45	1.28
EK - 2500 nM Ca^2+^ - 0 µM Ran	2.27	2.01	2.24	2.00
EK - 2500 nM Ca^2+^ - 10 µM Ran	0.84	0.58	0.81	0.57
**3 Hz-τ2 (s)**				
WT - 0 nM Ca^2+^ - 0 µM Ran	14.24	10.80	13.75	10.66
WT - 0 nM Ca^2+^ - 10 µM Ran	15.28	13.72	15.08	13.65
WT - 2500 nM Ca^2+^ - 0 µM Ran	39.52	40.39	39.63	40.43
WT - 2500 nM Ca^2+^ - 10 µM Ran	20.81	26.32	21.43	26.60
EK - 0 nM Ca^2+^ - 0 µM Ran	54.26	61.55	55.14	61.90
EK - 0 nM Ca^2+^ - 10 µM Ran	31.12	36.95	31.81	37.24
EK - 2500 nM Ca^2+^ - 0 µM Ran	43.81	68.26	46.41	69.75
EK - 2500 nM Ca^2+^ - 10 µM Ran	5.29	4.15	5.13	4.11

**Figure 9:**
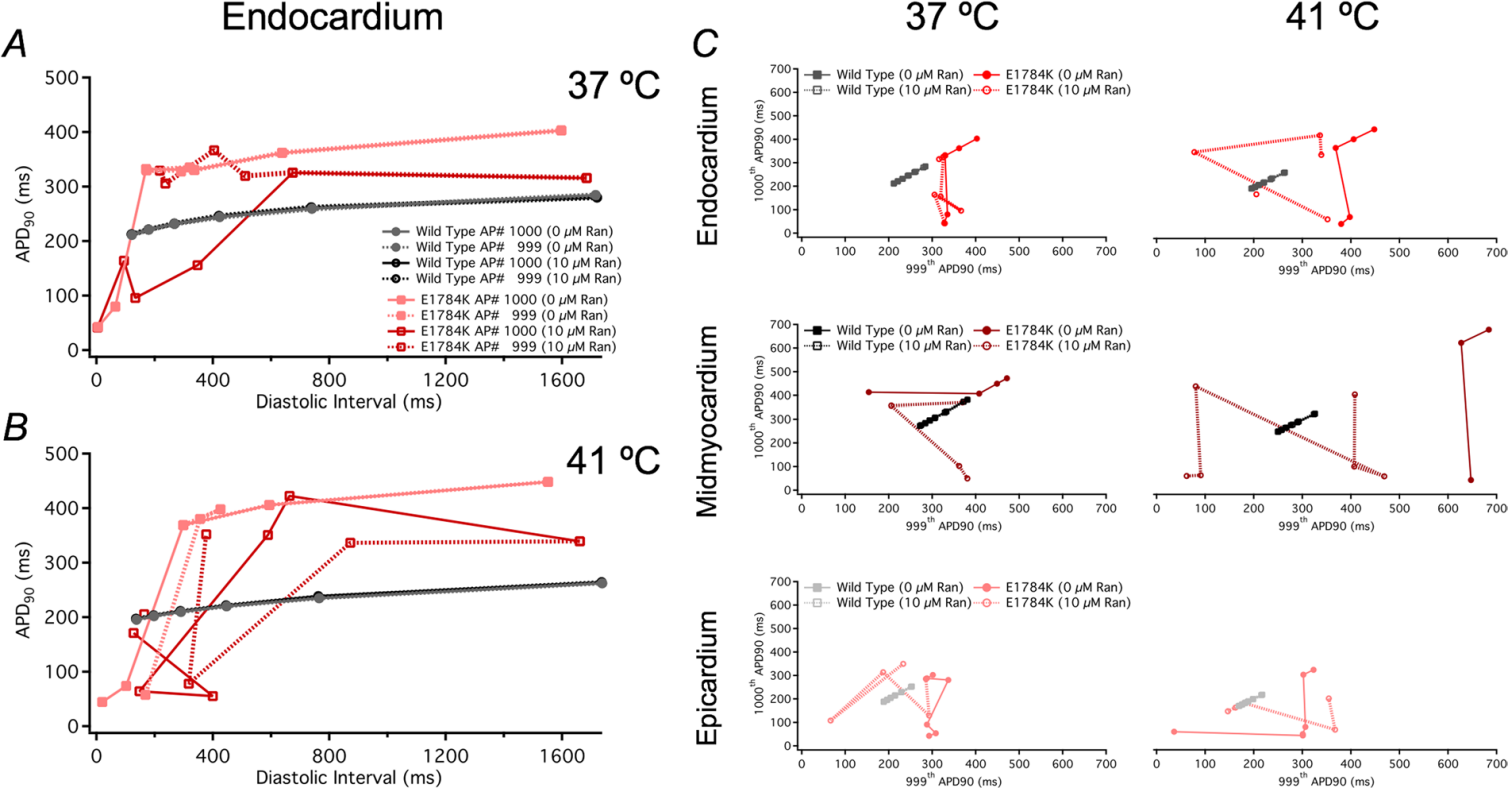
Cardiac electrical restitution properties. Panel A,B shows the endocardial ERC curves at 37 °C and 41 °C. Panels C shows plots of the last two AP beats to determine alternans-induction in the three myocardial cells at 37 °C and 41 °C.

Electrical restitution curves (ERCs) at 90% repolarization were constructed from plotting APD_90_ against the diastolic interval as shown in Fig. 9A,B in endocardial cells. The last two beats were included in the ERCs to exemplify bifurcation and alternans-induction at critical diastolic intervals. The APD_90_ for WT follows a similar trend to previously published ERCs, typifying a relatively stable APD rate dependence^56–58^. E1784K has a higher APD_90_ in all cardiac cells, especially the mid-myocardium, compared to WT (not shown in Fig. 9). At 37 °C, the mutant causes bifurcation in APD_90_, mainly in epicardial cells, indicative of alternans (not shown); however, endocardial cells also experience alternans at febrile temperature in addition to epicardial cells (Fig. 9B). Upon drug perfusion, bifurcations were observed at higher diastolic intervals in E1784K (Fig. 9). The drug-induced bifurcations in ERCs were augmented at febrile temperature in all cardiac cells.

A linear relationship is established between the last two AP beats at each frequency step (shown in Fig. 9C), with no alternans. Divergence from linearity is indicative of alternans occurrence. At both 37 °C and 41 °C, WT cells had no alternans, even upon 10 µM Ranolazine perfusion, showing linearity with a slope = 1 (Fig. 9C). In drug-free conditions, E1784K had a linear relationship at high BCLs (low frequencies), but deviated from linearity beginning at intermediate BCLs; distortion in linearity is observed at lower frequencies in epicardial cells (Fig. 9C). This relationship in E1784K is augmented with febrile temperature. The prolonged APD_90_ in E1784K were shortened with Ranolazine at very low frequencies, and alternans were quickly induced even during bradycardia, an effect exacerbated by febrile temperature (Fig. 9C).

## Discussion

Our goal was to determine whether Ranolazine reduced channel dysfunction in E1784K under the triggering conditions of elevated temperature and cytosolic calcium. Ranolazine did not attenuate gain-of-function in E1784K when temperature and cytosolic calcium were elevated. Ranolazine has minimal effects on conductance in Na_V_1.5^48,49^. The drug follows the modulated receptor hypothesis, targeting the open/inactivated states at depolarized potentials, thereby suppressing late I_Na_^59^. Physiological events, such as acidaemia, enhance Ranolazine antiarrhythmic effect by augmenting late I_Na_, thus providing the drug with a larger open-state channel substrate to target^48,60,61^. In addition to physiological modulators, *SCN5a* mutations often alter voltage-dependence of the channel, which modify drug effects on Na_V_1.5. To date, Ranolazine has been screened against only ∆KPQ^50,52^, Y1767C^49^, R1623Q^51^, and D1790G^62^. Our study is the first to show the combined external triggers and *SCN5a* mutation effects on Ranolazine.

Ranolazine efficacy was enhanced at elevated temperature. Similar to acidosis effects, elevated temperature increases the late open probability in E1784K^48,53^. Late I_Na_ percent and density increased by 11- and 7-fold, respectively, with elevated temperature. We previously reported a 3.54-fold increase in late I_Na_ percent when temperature was elevated from 22 °C to 34 °C^41^; however, we used CHOK1 cells to study E1784K thermosensitivity. The temperature coefficient (Q_10_) partly depends on lipid-channel interactions in the membrane, which differ between heterologous expression systems^63,64^. The HEK293 lipid bilayer is less viscous than CHOK1 cells as observed in our whole-cell recordings, which may justify the heightened Q_10_ in the late I_Na_ measurements. At elevated stimulation frequencies and temperature, therapeutic Ranolazine decreased channel availability by increasing channel use-dependence in E1784K.

Although Ranolazine efficacy appears to be increased by temperature, its efficacy appears to be dampened by the combination of both elevated temperature and cytosolic calcium. At 34 °C, E1784K late I_Na_ percent was depressed with elevated cytosolic calcium, but there was no effect on late I_Na_ density. These opposing changes in late I_Na_ percent and density may be attributed to the increased peak I_Na_ density with elevated cytosolic calcium at 34 °C. Although not significant, the shift contributes to late I_Na_ percent calculation (Table 1). Late I_Na_ in E1784K was not attenuated by Ranolazine at elevated temperature and cytosolic calcium.

### Drug Binding in Mutant-Trigger Context

Ranolazine action on Na_V_1.5 is commonly associated with the modulated receptor hypothesis^65^. Sokolov *et al*. argued that the drug follows a modified form of binding, as their pharmacological analysis suggested drug-binding at hyperpolarized voltages^48^. Ranolazine exhibits partial preference to the closed state in Na_V_1.5, as suggested by the hyperpolarizing shift in SSFI observed with Ranolazine. The shift mainly decreases channel availability near resting potential in cardiomyocytes. Other classic antiarrhythmics, anticonvulsants, and local anesthetics have similar effects, yet follow the modulated receptor hypothesis^59,66^. Sokolov *et al*. also report that the drug-induced block in late I_Na_ occurs by a slow-mode recovery in slow inactivation, which is exacerbated at low pH^48^. This effect was mildly observed in this study. However, it is difficult to correlate the drug effects in E1784K UDI to late I_Na_, as the former had minor but significant shifts.

Ranolazine shares a very similar structure with the class 1b antiarrhythmic drug lidocaine, which, like other sodium blockers, preferentially binds to F1760 and, to a lesser extent, Y1767 in DIVS6^49,52^. Ranolazine has high lipophilicity and can only bind to its receptor sites by traversing the phospholipid membrane and entering the central cavity through the inner vestibule. However, lateral pores, known as fenestrations, are alternative routes for large compounds like Ranolazine to access the central cavity. The fenestrations in the Na_V_Pas-Na_V_1.5 model were unavailable for drug binding in auto-docking due to their constricted sizes^67^. It would be interesting to determine whether E1784K alters fenestration size in Na_V_1.5, modifying drug entry via the fenestrations.

The interaction between Ranolazine and extracellular channel regions is unknown, but is unlikely due to its lipophilicity; a crystal structure of the channel/drug interaction would elucidate the drug-induced modifications in gating. The newly discovered aryl sulfonamide antagonists preferentially stabilize Na_V_1.7 DIVS4 activation thereby stabilizing the fast inactivated state and suppressing late I_Na_^68^. Ranolazine may be structurally modified to include other moieties, like anionic aryl sulfonamides, for further optimizing its selectivity for targeting late versus peak I_Na_.

### E1784K-induced Structural Rearrangements in NaV1.5 and its Impact on Ranolazine

We speculate E1784K affects fast inactivation via two possible mechanisms in Na_V_1.5, thereby altering drug-channel interactions. Figure 1 shows the channel structures discussed, as follows:

1. E1784K hyperpolarizes the voltage-dependence of SSFI, thus stabilizing the interaction between the channel and the fast inactivation particle^7,11,12,22,28^. Fast inactivation onset is correlated with DIVS4 activation, whereas channel recovery is rate limited by charge immobilization of DIVS4^69,70^. The charge reversal mutant, E1784K, may enhance the transition of DIVS4 between closed and open states, as suggested by the Peters-Ruben model^43^. We postulate that this effect may be due to an electrostatic repulsion between the CTD mutant and conserved positive residues in DIVS4, given their close proximity^67^. This repulsion could make the DIVS4 in E1784K more mobile, which might explain the accelerated fast inactivation onset and recovery kinetics^22,37,41,43^. Fast inactivation kinetics in E1784K are not enhanced by temperature, so it does not seem justified to attribute the thermosensitive late I_Na_ in E1784K to increased recovery kinetics. Rather a rearrangement may occur in DIVS4, conforming the voltage sensor to a state in which conductance in E1784K is higher^43^.
2. E1784K alters the structure of CTD by disrupting the native hydrophobic and electrostatic interactions holding the EF-like hand domain (α_1_–α_4_) tight with the IQ motif (α_6_)^31^. Calcium sensitivity is imparted in Na_V_1.5 via CaM, which binds to the IQ motif (α_6_) via its C-lobe or N-lobe depending on cytosolic calcium levels^71–73^. During diastole or systole, CaM is calcified to different extents at its N-lobe^74^. Calcified CaM has a lower affinity for the IQ motif and binds, via its C-lobe, to DIII-DIV linker, forming a tripartite complex. This interaction is thought to prevent the DIII-DIV linker fast inactivation particle from occluding the pore, increasing channel availability near resting potential^71,73^. With depolarized potentials, the CaM C-lobe stabilizes the fast inactivation particle, suppressing late I_Na_, as in ∆KPQ and other mixed syndrome mutants^37,40,44^. Some studies refute the tripartite complex formation and favor a Ca_V_1.2-like regulation of inactivation in Na_V_1.5^74,75^. In those studies, the calcium-calmodulin complex is localized to CTD^76,77^. The Na_V_Pas structure showed intermolecular interactions between DIII-DIV linker, CTD, and DIVS4^67^. Motoike *et al*. reported CaM-independent interactions between the inter-linkers in Na_V_1.5^36^, suggesting that CaM acts as an auxiliary channel modifier during a calcium signal^75^. Calcium regulation in Na_V_1.5 is mediated by CaM since the dual EF-like hand domains in CTD do not bind calcium^74,75^. In light of these structural models, we speculate that E1784K decouples both the calcium-dependent and calcium–independent interactions between the DIII-DIV linker and the CTD. Thus, E1784K inhibits calcium-dependent facilitation in Na_V_1.5. We propose that the decoupling in CTD caused by E1784K may create a high entropy, unstable structure. Upon a calcium signal, the calcified calmodulin has reduced affinity for the IQ motif, thus augmenting CTD entropy^73^.

Both mechanisms **(**1**)** and (2) may occur simultaneously in E1784K. The calcium effects in Na_V_1.5 are localized to CTD. No reports have shown direct interaction between calcium-calmodulin and DIVS4, so if mechanism (2) occurs, it may be via an indirect effect on DIVS4.

In light of the discussed structural insights, we speculate that Ranolazine can easily access the inner vestibule with non-calcified calmodulin, since the molecule binds tightly to the IQ motif. Ranolazine efficacy, however, is hampered by cytosolic calcium, suggesting an interaction between the drug and the channel at CTD. The high entropy CTD in calcified calmodulin seems to physically hinder Ranolazine from entering into the inner vestibule.

### Physiological and Medical Implications

Elevated temperature and cytosolic calcium are two of many other physiological triggers that occur during exercise and are common to other pathophysiological states, such as myocardial ischemia or infarction, and heart failure^78,79^. The majority patients with *SCN5a* mutations show ameliorated LQT3 phenotype during exercise^80^. Functional studies have correlated this to a stimulation frequency or calcium-induced reduction in late I_Na_^44^. However, it is clear from our study, focusing on E1784K, that the *SCN5a* mutant response to triggers can be unique^37,41^. Thus, it is necessary to study antiarrhythmics in *SCN5a* cohorts during different physiological states as the mutant-trigger effect may determine drug efficacy.

Our AP simulations clearly show pro-arrhythmic effects of Ranolazine, which are exacerbated by febrile temperatures. Electrical restitution curves clearly show a critical diastolic interval at which alternans are triggered. Our AP simulations provide evidence of Ranolazine’s arrhythmogenicity, as it does not shorten APD_90_ in E1784K at high heart rates. At low heart rates and at body core temperature, the drug shortens APD_90_ in cardiac cells. However, with normal and elevated heart rates, the drug induces alternans, an effect exacerbated at febrile temperature. The critical diastolic intervals at which alternans are caused by the drug appear earlier (at higher BCLs) at febrile temperature.

E1784K induces alternans with higher prevalence in epicardial cells at low heart rates. This result coincides with the phase 2 re-entry phenomenon constituting the repolarization hypothesis in BrS1^78^. The high I_Kto_ density, especially in the right epicardium, results in complete action potential failure^81^. E1784K channels are less available for activation due to the hyperpolarized SSFI-V_1/2_. This seems to be the main mechanism behind the decrease in AP upstroke velocity in cardiac cells, especially the epicardial cells, despite the mutant and triggers-exacerbated increased late I_Na_. Thus, E1784K expresses both gain- and loss-of-function at the electrical level in cardiac cells. However, this expressivity is finely tuned by channel switches, like temperature and cytosolic calcium.

Our previous and current data suggest exercise, and its accompanying physiological triggers, differentially affect mixed syndrome mutations, especially E1784K^22,37,41,42^. The action of different antiarrhythmics appear to differ depending on physiological state.

## Conclusions

Appropriate management of cardiac arrhythmias in *SCN5a* patients requires careful investigation of antiarrhythmic drug efficacy under various physiological states. Our results suggest that Ranolazine may increase the susceptibility for arrhythmia development in E1784K carriers at sinus rhythm and tachycardia. The risk is augmented under febrile conditions. Although exercise is commonly associated with high heart rates, other pathophysiological states share common triggers, as in heart failure or myocardial ischemia and infarction. Other antiarrhythmics should also be screened against E1784K and other channel mutants under various physiological conditions.

## Methods

### Homology Modelling and Auto-Docking

Homology modeling was performed using the Swiss-Model server (https://swissmodel.expasy.org)^82^. The newly cryo-EM solved American cockroach voltage-gated sodium channel (Na_V_Pas) structure (3.8-Å resolution) was used as a template against the Na_V_1.5 sequence. Modeling was done according to the protocol established by Bordoli *et al*.^82^. Sequence alignment was performed using Uniprot Align (http://www.uniprot.org/align/) for SCN5A_HUMAN (Na_V_1.5) and SCNA1_PERAM (Na_V_Pas).

Ranolazine was virtually docked using AutoDock4 against the Na_V_1.5 homology model built on Na_V_Pas (Na_V_1.5-Na_V_Pas)^83^. PyMOL-pdb viewer was used for optimization and visualization of the auto-docking results.

### Ethical approval

The research was approved by Biohazards review 251–2012 issued by the office of the Environmental Health and Safety at Simon Fraser University, Burnaby, BC, Canada.

### Cell Culture

HEK293 cells were grown at pH 7.4 in a DMEM (1x) nutrient medium (Life Technologies, NY, USA), supplemented with 10% FBS and maintained in a humidified environment at 37 °C with 5% CO_2_. The α subunits (WT or E1784K) were co-transfected with the β1 subunit and green fluorescent protein, eGFP (1.50 µg: 0.75 µg: 1.50 µg, respectively). The cDNA mixture was then allowed to incubate with the HEK293 cells before plating on coverslips. The HEK293 cells were selected for this study since they contain a relatively elevated [CaM]_free_ level compared to other cell lines, thereby controlling for calcium-calmodulin effects on Na_V_1.5^84^.

### Electrophysiology

Whole-cell patch clamp recordings were performed in extracellular solution containing (mM): 96 NaCl, 4 KCl, 2 CaCl_2_, 1 MgCl_2_, and 10 HEPES (pH 7.4). Solutions were titrated with CsOH to pH 7.4. Pipettes were fabricated with a P-1000 puller using borosilicate glass (Sutter Instruments, CA, USA), dipped in dental wax to reduce capacitance, then thermally polished to a resistance of 1.0–1.5 MΩ. Low resistance electrodes were used to minimize series resistance between pipette and intracellular solution resulting in typical access resistances of 3.5 MΩ or less, thereby minimizing voltage measurement error. Pipettes were filled with intracellular solution. For minimal cytosolic calcium levels, pipettes contained (mM): 130 CsF, 9.6 NaCl, 10 HEPES, and 10 EGTA titrated to pH 7.4. The intracellular pipette solution was manipulated to mimic peak systolic cytosolic calcium^85,86^. To do so, we calculated, using the Ca-EGTA Calculator v1.3, the amount of CaCl_2_ (in mM) added to bring cytosolic calcium to 2500 nM at both 22 °C and 34 °C: 9.53 and 9.60, respectively.

All recordings were made using an EPC-9 patch-clamp amplifier (HEKA Elektronik, Lambrecht, Germany) digitized at 20 kHz using an ITC-16 interface (HEKA Elektronik, Lambrecht, Germany). Data were acquired and low-pass-filtered (5 kHz) using PatchMaster/FitMaster software (HEKA Elektronik, Lambrecht, Germany) running on an Apple iMac (Apple Computer, Cupertino, CA). Leak subtraction was performed online using a P/4 procedure. Bath solution temperature was controlled using a Peltier device driven by a TC-10 Temperature Controller (Dagan, Minneapolis, MN). Bath temperature was maintained at 22 °C or 34 °C. Experiments were not performed at physiological temperatures because of the inherent instability of cells at temperatures above 34 °C. We extrapolated data to physiological temperatures using a Q_10_ relationship, which was supported with Arrhenius calculations, (described below). After a giga ohm seal resistance was achieved, the whole-cell configuration was attained. The holding potential between protocols was −110 mV. We recorded I_Na_ from cells that expressed currents no greater than −5 nA. The average voltage error calculated for all cells used in this study (n = 250) is 6.06 mV ± 0.40 mV obtained (Table 7). There are no differences between the voltage-errors in the different conditions (p > 0.05).

**Table 7:** Voltage Error.

**Condition**	**Voltage Error (mV)**	**N**
WT - 22 °C - 0 nM Ca^2+^ - 0 µM Ran	3.52 ± 0.97	9
WT - 22 °C - 0 nM Ca^2+^ - 10 µM Ran	7.83 ± 1.47	8
WT - 22 °C - 0 nM Ca^2+^ - 100 µM Ran	5.70 ± 2.05	6
WT - 22 °C - 2500 nM Ca^2+^ - 0 µM Ran	4.89 ± 1.29	9
WT - 22 °C - 2500 nM Ca^2+^ - 10 µM Ran	6.54 ± 1.30	11
WT - 22 °C - 2500 nM Ca^2+^ - 100 µM Ran	3.12 ± 0.88	10
WT - 34 °C - 0 nM Ca^2+^ - 0 µM Ran	8.35 ± 1.56	11
WT - 34 °C - 0 nM Ca^2+^ - 10 µM Ran	9.08 ± 1.32	9
WT - 34 °C - 0 nM Ca^2+^ - 100 µM Ran	7.18 ± 1.16	19
WT - 34 °C - 2500 nM Ca^2+^ - 0 µM Ran	8.64 ± 0.98	7
WT - 34 °C - 2500 nM Ca^2+^ - 10 µM Ran	8.53 ± 1.79	9
WT - 34 °C - 2500 nM Ca^2+^ - 100 µM Ran	8.14 ± 1.57	6
EK - 22 °C - 0 nM Ca^2+^ - 0 µM Ran	4.41 ± 0.65	19
EK - 22 °C - 0 nM Ca^2+^ - 10 µM Ran	5.99 ± 1.22	10
EK - 22 °C - 0 nM Ca^2+^ - 100 µM Ran	4.28 ± 0.72	14
EK - 22 °C - 2500 nM Ca^2+^ - 0 µM Ran	3.01 ± 0.71	6
EK - 22 °C - 2500 nM Ca^2+^ - 10 µM Ran	4.49 ± 1.04	9
EK - 22 °C - 2500 nM Ca^2+^ - 100 µM Ran	4.63 ± 1.21	10
EK–34 °C - 0 nM Ca^2+^ - 0 µM Ran	6.50 ± 1.09	19
EK - 34 °C - 0 nM Ca^2+^ - 10 µM Ran	6.74 ± 1.73	10
EK - 34 °C - 0 nM Ca^2+^ - 100 µM Ran	5.52 ± 0.98	12
EK - 34 °C - 2500 nM Ca^2+^ - 0 µM Ran	5.63 ± 1.32	8
EK - 34 °C - 2500 nM Ca^2+^ - 10 µM Ran	8.95 ± 1.97	8
EK - 34 °C - 2500 nM Ca^2+^ - 100 µM Ran	3.67 ± 0.69	11

### Drug Preparation

Ranolazine was obtained from Gilead Sciences (Foster City, CA) in powder form, diluted to 100 mM stock in 0.1 M HCl, aliquoted at 10 mM and stored at −20 °C. Working concentrations of 10 µM (therapeutic concentration) or 100 μM (non-therapeutic) were freshly prepared in bath solution. pH was readjusted before performing electrophysiological experiments. Due to the large number of experimental conditions and the challenges of maintaining whole-cell recordings at elevated temperature, we performed unmatched pair experiments.

### Analysis and Statistics

Analysis and graphing were done using FitMaster software (HEKA Elektronik, Lambrecht, Germany) and Igor Pro (Wavemetrics, Lake Oswego, OR, USA) with statistical information derived using JMP statistical software. Statistical significance was accepted at p < 0.05 using a four-factor completely randomized design (CRD) ANOVA test followed by a post-hoc Tukey test. Our statistical model was a full factorial in which all the factors were allowed to interact together yielding multiple effect tests: Ranolazine, Channel Variant, Ranolazine × Channel Variant, Temperature, Ranolazine × Temperature, Channel Variant × Temperature, Ranolazine × Channel Variant × Temperature, Calcium, Ranolazine × Calcium, Channel Variant × Calcium, Ranolazine × Channel Variant × Calcium, Temperature × Calcium, Ranolazine × Temperature × Calcium, Channel Variant × Temperature × Calcium, Ranolazine × Channel Variant × Temperature × Calcium. All values reported in the results sections are given as means ± standard error of means.

### Voltage Protocols

#### Current Density

We measured current density from the ratio of current amplitude to the cell membrane capacitance (pA/pF).

#### Conductance Density

Channel conductance was calculated from peak I_Na_ using Ohm’s law at 0 mV.

1 $${{\rm{G}}}_{{\rm{Na}}}={{\rm{I}}}_{{\rm{Na}}}/{\rm{V}}-{{\rm{E}}}_{{\rm{rev}}}$$

where G_Na_ is sodium channel conductance, I_Na_ is peak sodium current in response to the command potential V = 0 mV, and E_rev_ is the reversal potential. We measured conductance density from the ratio of conductance to the cell membrane capacitance (nS/pF).

#### Activation (GV)

To determine the voltage dependence of activation, we measured the peak current amplitude at test pulse potentials ranging from −100 mV to +80 mV in increments of +10 mV for 19 ms. Prior to the test pulse, channels were allowed to recover from fast inactivation at −130 mV for 197 ms. Channel conductance was calculated from peak I_Na_ using Formula **(1)**. Calculated values for conductance were normalized to the maximal conductance and fit with the Boltzmann function:

2 $${\rm{G}}/{{\rm{G}}}_{{\rm{\max }}}=1/(1+\exp [{-\text{ze}}_{0}[{{\rm{V}}}_{{\rm{m}}}-{{\rm{V}}}_{1/2}]/\text{kT}])$$

where G/G _max_ is the normalized conductance amplitude, V_m_ is the command potential, z is the apparent valence, e_0_ is the elementary charge, V_1/2_ is the midpoint voltage, k is the Boltzmann constant, and T is temperature in °K.

#### Steady-State Fast Inactivation (SSFI)

The voltage-dependence of SSFI was measured by preconditioning the channels to a hyperpolarizing potential of −130 mV and then eliciting prepulses from −130 or −150 to +10 mV in increments of 10 mV for 500 ms. Channel availability was assessed during a test pulse to 0 mV. Normalized current amplitude as a function of voltage was fit using the Boltzmann function:

3 $${\rm{I}}/{{\rm{I}}}_{{\rm{\max }}}=1/(1+\exp (-{{\rm{ze}}}_{0}({{\rm{V}}}_{{\rm{M}}}-{{\rm{V}}}_{1/2})/{\rm{kT}})$$

where I/I_max_ is the normalized current amplitude, z is apparent valence, e_0_ is the elementary charge, V_m_ is the prepulse potential, V_1/2_ is the midpoint voltage of SSFI, k is the Boltzmann constant, and T is temperature in °K.

#### Fast Inactivation Onset

Time constants for open-state fast inactivation were derived by fitting a single exponential function to the decay of current obtained from the activation protocol.

4 $${\rm{I}}={{\rm{I}}}_{{\rm{ss}}}+{\rm{\alpha }}\exp (-(t-{{\rm{t}}}_{0})/{\rm{\tau }})$$

where I is current amplitude, I_ss_ is the plateau amplitude, α is the amplitude at time 0 for time constant τ, and t is time.

#### Late I_Na_ Current

Late I_Na_ was measured between 40–50 ms during a 50 ms depolarizing pulse to 0 mV from a holding potential of −130 mV. An average of 10 pulses was used to increase the signal-to-noise ratio.

#### Use-Dependent Inactivation (UDI, 1 Hz and 3 Hz)

Channels accumulated into a use-dependent inactivated state during either a series of 300 380 ms depolarizing pulses to 0 mV followed by a 615 ms–110 mV recovery pulse at a frequency 1 Hz, or 500 220 ms depolarizing pulses to 0 mV followed by a 110 ms–110 mV recovery pulse at a frequency 3 Hz. Normalized current amplitude as a function of time was fit with a double exponential.

5 $${\rm{I}}={{\rm{I}}}_{{\rm{ss}}}+{{\rm{\alpha }}}_{1}\exp (-{\rm{t}}/{{\rm{\tau }}}_{1})+{{\rm{\alpha }}}_{2}\exp (-{\rm{t}}/{{\rm{\tau }}}_{2})$$

where I is current amplitude, I_ss_ is the plateau amplitude, α_1_ and α_2_ are the amplitudes at time 0 for time constants τ_1_ and τ_2_, and t is time.

#### Q_10_ Coefficients

The temperature coefficient for kinetic and thermodynamic parameters plotted as a function temperature was calculated in Igor:

6 $${{\rm{Q}}}_{10}=({{\rm{R}}}_{2}/{{\rm{R}}}_{1}){}^{10/(\text{T2}\mbox{--}\text{T1})}$$

where R is the rate and T is temperature (1 and 2 are the two temperatures measured). Rate was calculated by the inverse of the τ value. Q_10_ fits for steady-state midpoints and slopes were calculated by replacing the R_X_ with V_1/2_ and z values. Fits for y_0_ were calculated based of the 1/y_0_ to yield optimal Q_10_ values. The fit was extrapolated to physiological (37 °C) and febrile (41 °C) temperatures.

#### Arrhenius Calculations

The Arrhenius linear relationship for the natural exponent of kinetic or thermodynamic parameters as a function of inverse temperature was calculated in Igor:

7 $$\mathrm{ln}({\rm{k}})=\,\mathrm{ln}({\rm{A}})-({\rm{Ea}}/{\rm{R}})\times (1/{\rm{T}})$$

where k is the rate constant, steady-state midpoint, or slope, A is the pre-exponential factor, Ea is the activation energy, R is the universal gas constant, and T is temperature in °K.

### Myocardial Action Potential (AP) Modeling

#### Simulations

Action potentials were simulated using a modified version of the O’Hara Rudy (ORd) model at 37 °C and 41 °C programmed in Matlab^87^. The sodium data was extrapolated to physiological and febrile temperatures Q_10_ values for WT and E1784K at 0 µM and 10 µM Ranolazine. The maximal G_Na_ density was 150 mS/µF in all conditions simulated. We modified the gating I_Na_ parameters data in accordance with our biophysical data for the various conditions. The GV and SSFI midpoints and slopes were extrapolated to 37 °C and 41 °C and normalized to the original ORd parameters. The phosphorylated steady-state fast inactivation midpoints in all channel variants were equally hyperpolarized by 6.2 mV. Late I_Na_ density was normalized to the original ORd value and multiplied by the percentage of late to peak I_Na_ calculated above.

To model the calcium-dependence of our late I_Na_ data, we fit the biophysical parameters extrapolated to 37 °C and 41 °C with a Hill equation:

8 $${\rm{Z}}={{\rm{Y}}}_{0}+{(Y}_{{\rm{M}}}-{{\rm{Y}}}_{0})/(1+{({{\rm{X}}}_{1/2}/{\rm{X}})}^{{\rm{b}}})$$

where Z is the biophysical parameter of interest, Y_0_ is the minimum value, Y_M_ is the maximum value, X_1/2_ is the midpoint of the curve, X is the intracellular cytosolic calcium, b is the rate.

Subspace calcium was not accounted for due to the lack of experimental data. Thus, the modified ORd model is a dynamic simulation of the calcium-induced shifts which are observed with increasing intracellular calcium levels as a function of pacing frequency, comprising the positive staircase phenomenon^88,89^.

Simulations at febrile temperature (41 °C) included modifications to the major ionic currents, I_Kto_^90^, I_CaL_^91,92^, I_Kr_^93^, and I_Ks_^94^, in the ORd model based on previously published Q_10_ values.

Simulations were run on endocardial, midmyocardial, and epicardial ventricular myocytes using a 0.5 ms stimulus pulse with an amplitude of −80 µA/µF. The stimulus protocol was designed to step up the frequency gradually from 0.5 Hz to 2.5 Hz, with a 1000 beats per frequency step to ensure attainment of steady-state.

#### Analysis

Analysis of APs only included those that fully recovered and were restored to baseline. Action potential duration (APD) was measured at 90% of repolarization by multiplying the resting membrane potential (RMP) value, prior to the current stimulus pulse, by 0.9. The APD_90_ of the final two beats in the frequency step were plotted versus the diastolic interval (DI = BCL − APD_90_), where BCL is the basic cycle length, creating electrical restitution curves.

#### Data Availability

All data generated or analysed during this study are included in this published article.
